# Type IV secretion systems: reconciling diversity through a unified nomenclature

**DOI:** 10.1093/femsre/fuaf069

**Published:** 2025-12-31

**Authors:** Peter J Christie, Gabriel Waksman, Ronnie Per-Arne Berntsson, Nicolas Soler, Nathalie Leblond-Bourget, Badreddine Douzi

**Affiliations:** Department of Microbiology and Molecular Genetics, McGovern Medical School, UTHealth Houston, Houston, TX, 77030, United States; Institute of Structural and Molecular Biology, School of Natural Sciences, Birkbeck College, Malet Street, London WC1E 7HX, United Kingdom; Institute of Structural and Molecular Biology, Division of Biosciences, Gower Street, University College London, London WC1E 6BT, United Kingdom; Department of Medical Biochemistry and Biophysics, Umeå University, Wallenberg Centre for Molecular Medicine & Umeå Centre for Microbial Research, Umeå University, SE-90187 Umeå, Sweden; Université de Lorraine, INRAE, DynAMic, F-54000 Nancy, France; Université de Lorraine, INRAE, DynAMic, F-54000 Nancy, France; Université de Lorraine, INRAE, DynAMic, F-54000 Nancy, France

**Keywords:** Type IV secretion systems (T4SSs), horizontal gene transfer, conjugation, effectors secretion, relaxosome, nomenclature

## Abstract

Type IV secretion systems (T4SS) are versatile nanomachines responsible for the transfer of DNA and proteins across cell envelopes. From their ancestral role in conjugation, these systems have diversified into a superfamily with functions ranging from horizontal gene transfer to the delivery of toxins to eukaryotic and prokaryotic hosts. Recent structural and functional studies have uncovered unexpected architectural variations not only among Gram-negative systems but also between Gram-negative and Gram-positive systems. Despite this diversity, a conserved set of core proteins is maintained across the superfamily. To facilitate cross-system comparisons, we propose in this review a unified nomenclature for conserved T4SS subunits found in both Gram-negative and Gram-positive systems. We further highlight conserved and divergent mechanistic and architectural principles across bacterial lineages, and we discuss the diversity of emerging T4SSs whose unique structures and functions expand our understanding of this highly adaptable secretion superfamily.

## Teaser

This review presents a comprehensive overview of Type IV Secretion Systems (T4SSs), focusing on their structural, functional, and evolutionary diversity across bacterial lineages. With recent advances in cryo-EM and cryo-ET revealing numerous T4SS structures and subcomplexes, a unified nomenclature is increasingly necessary for effective comparison and interpretation. The review aims to establish standardized terminology for conserved T4SS components, including Type IV coupling and Relaxosomal proteins shared between Gram-negative and Gram-positive bacteria. It further compares mechanistic and architectural principles across these groups and highlights emerging, understudied T4SSs that broaden understanding of this adaptable secretion machinery.

## Introduction

The discovery of interbacterial genetic recombination by Lederberg and Tatum (1946) established the principle of bacterial sex or conjugation, whereby donor cells deliver mobile genetic elements (MGEs) to recipient cells through a mechanism generally requiring direct cell-to-cell contact. In the ensuing decades, the F plasmid and many other conjugative plasmids emerged as important models for defining the mechanistic and structural features of conjugation (Schroder and Lanka 2005, Arutyunov and Frost 2013, Cabezon et al. 2014, Waksman 2019). Invariably, these studies established that transmission of all MGEs requires three sets of genes with distinct functions. The *dtr* (DNA transfer and replication) genes code for proteins involved in processing the MGE for transfer (de la Cruz et al. 2010). They include the catalytically active relaxase and one or more auxiliary proteins; these proteins assemble as the relaxosomal complex at the MGE’s origin-of-transfer (*oriT*) sequence to nick the DNA strand (T-strand) destined for transfer (Williams et al. 2025). A larger set of *MPF* (mating pair formation) genes code for the subunits of a cell-envelope-spanning machine and sometimes an extracellular conjugative pilus; this machine conveys the processed DNA substrate from the donor to a recipient cell (Costa et al. 2023, Breidenstein et al. 2025). In the mid-1990’s, MPF machines were renamed as the Type IV Secretion Systems (Salmond 1994, Christie 1997). The third set consists of one gene that encodes the receptor for cognate DNA substrates; this receptor physically couples the DNA substrate to the T4SS machine and hence is termed the T4CP (Type IV Coupling Protein) (Cabezon et al. 1997, Gomis-Ruth et al. 2002). Further investigations of some T4CPs, most notably that of *Legionella*, supplied evidence that the coupling protein is part of a larger complex, now termed the Type IV coupling complex (T4CC), which serves as a general substrate-recruitment machinery powered by the T4CP (Meir et al. 2020).

Over evolutionary time, ancestral conjugation systems were functionally repurposed to deliver DNA substrates into the extracellular milieu, take up exogenous DNA through a mechanism akin to transformation, or conjugatively deliver toxic protein substrates to kill other bacteria for niche establishment (Alvarez-Martinez and Christie 2009, Souza et al. 2015). With the appearance of eukaryotic cells, conjugation systems were further adapted to deliver effector proteins instead of or in addition to DNA substrates (Cascales and Christie 2003). While most systems deliver effector proteins directly into eukaryotic cells to aid in establishment of symbiotic or pathogenic relationships, at least one evolved the capacity to export a multisubunit toxin to the extracellular milieu. These protein translocation systems, which comprise a functionally distinct subfamily of T4SSs from that of the conjugation systems, are now termed the effector translocators. Many effector translocator systems are assembled only from homologs of the core set of subunits required for conjugative DNA transfer; collectively with ancestral conjugation systems, these are termed “minimized” T4SSs (Bhatty et al. 2013, Costa et al. 2021). However, in addition to the set of core subunits, many other effector translocators as well as some conjugation systems have appropriated additional subunits from unknown ancestries, presumably endowing the systems with specialized functions; these systems are now grouped as the “expanded” T4SSs (Costa et al. 2021). For the “expanded” T4SSs, the gene nomenclatures are highly system-specific and bear no resemblance to nomenclatures adopted for other conjugation or effector translocator systems (Costa et al. 2021). To facilitate comparisons of T4SSs, it is important to refer to machine subunits with common functions or structures that are conserved among the different systems. One major goal of this review is to present a unified nomenclature for the highly-conserved T4SS genes/proteins.

Remarkably, recent advances in structural definition of a few model T4SSs have revealed features of these machines that were unexpected based on prior biochemical and genetic studies. For example, as discussed further below, three machine subunits that are highly conserved among all T4SSs functioning in Gram-negative bacteria invariably assemble as intrinsically stable, outer membrane core complexes (OMCCs). However, OMCCs can adopt very different architectures and subunit stoichiometries, the latter translating to different fold symmetries among the structural subassemblies (Costa et al. 2023). Additionally, while there is accumulating evidence that conjugation systems of the single-membrane (monoderm) Gram-positive bacteria possess features that are conserved among the machines operating in double-membrane (diderm) Gram-negative bacteria, the Gram-positive systems also have evolved significant structural adaptations (Breidenstein et al. 2025). A second goal of this review is to highlight mechanistic and architectural themes and variations among T4SSs functioning in Gram-negative vs Gram-positive species. Finally, to date only a few model T4SSs have been extensively characterized, and yet a hallmark feature of the T4SS superfamily is their extensive functional diversity (Cascales and Christie 2003, Grohmann et al. 2018, Costa et al. 2021). Detailed studies of such diverse systems are needed to generate a full picture of the types of distinct structural motifs evolved by members of the T4SS superfamily and how these adaptations confer novel functions. In a final section of this review, we describe emerging or previously known but understudied T4SSs whose novel functions or architectural features merit further interrogation.

## The VirB/D4 nomenclature: a short history

We propose unifying nomenclatures for the MPF and Dtr proteins associated with conjugation systems and other T4SSs. The nomenclature for Dtr proteins is described in more detail below; here, we present a short history underlying the nomenclature proposed for the conserved subunits of the cell-envelope-spanning T4SS channels. This nomenclature arose from a discovery in the late 1970’s that *Agrobacterium tumefaciens* delivers oncogenic T-DNA to plant cells, resulting in a tumorous disease called Crown Gall (Chilton et al. 1977). In an autobiography, Eugene Nester describes this exciting finding and follow-up studies establishing that *A. tumefaciens* deploys a conjugation machine for interkingdom T-DNA transfer (Nester 2014). One important development involved transposon mutagenesis and genetic complementation analyses showing that a virulence region (Vir) harbored by the tumor-inducing (Ti) plasmid consisted of six complementation groups: *virA, virB, virC, virD, virE*, and *virG* (Iyer et al. 1982, Klee et al. 1982, Klee et al. 1983, Stachel and Nester 1986). In 1966, Demerec and colleagues, proposed a uniform nomenclature for bacterial genes in which three lower case letters indicate the pathway or process in which the gene product is involved and a capital letter signifies the actual gene, and all letters are italicized (Demerec et al. 1966). For the Vir region, sequence analyses ultimately confirmed predictions that the complementation groups consisted of transcriptional units, but only the *virA* and *virG* complementation groups consisted of single genes (Winans et al. 1986, Melchers et al. 1987) and thus adhered to the mnemonic proposed by Demerec et al. (Demerec et al. 1966). As the other complementation groups encompass multiple genes, numbers were assigned to each gene to denote its identity and order within the transcriptional unit, e.g. the *virB* unit consists of 11 genes (*virB1-11*) (Ward et al. 1988, Kuldau et al. 1990, Shirasu et al. 1990, Beijersbergen et al. 1994). Besides identifying genes within the transcriptional units, sequence analyses shed light on the possible contributions of each set of genes to the infection process. Most importantly for the T4SS field, most of the 11 *virB* genes encode proteins predicted to integrate into the cytoplasmic membrane or be exported to the periplasm (Ward et al. 1988, Kuldau et al. 1990, Shirasu et al. 1990, Beijersbergen et al. 1994). This prompted speculation that the VirB proteins form the channel through which the oncogenic T-DNA is delivered across the *A. tumefaciens* cell envelope to plant cells. Further studies established that the 5′ end of the *virD* unit encodes two proteins, one of which possesses endonuclease activity capable of site- and strand-specific cleavage of T-DNA border sequences (Yanofsky et al. 1986, Albright et al. 1987, Stachel et al. 1987, Wang et al. 1987). Researchers recognized parallels between the functions proposed for the VirB and VirD proteins and similar functions associated with the F plasmid and other conjugation systems (Stachel and Zambryski 1986, Christie et al. 1988, Shirasu and Kado 1993). *Agrobacterium tumefaciens* was thus envisioned to have appropriated an ancestral conjugation system for the novel purpose of delivering oncogenic T-DNA to plant cells to incite crown gall disease. Indeed, further sequence comparisons ultimately confirmed that the VirB proteins are homologs of plasmid-encoded MPF proteins implicated in assembly of the DNA transfer channel, and VirD1 and VirD2 are homologs of Dtr proteins, specifically the auxiliary proteins and relaxases shown to bind and nick *oriT* sequences to initiate plasmid transfer (Yanofsky et al. 1986, Albright et al. 1987, Lessl et al. 1992). Another protein encoded from within the *virD* transcriptional unit, VirD4, was also of considerable interest. It was not only shown to be essential for T-DNA transfer (Stachel and Nester 1986, Lin and Kado 1993), but sequence comparisons established it to be a homolog of plasmid-encoded proteins implicated in coupling DNA substrates to the translocation apparatus (Ziegelin et al. 1991, Llosa et al. 1994).

That *A. tumefaciens* adapted an ancestral conjugation system for interkingdom DNA transfer was further substantiated by findings that the T-DNA transfer system also mobilizes nonself-transmissible plasmids to plant cells (Buchanan-Wollaston et al. 1987). These findings establish that the Dtr factors and *oriT* sequences carried by the mobilizable plasmids are not only the functional equivalents of the VirD processing proteins and T-DNA border sequences, respectively, but also that the processed plasmids can be recognized as *bona fide* substrates of the T-DNA transfer system. Indeed, in an *A. tumefaciens* donor carrying both the mobilizable plasmid and T-DNA substrates, the plasmid was shown to outcompete the T-DNA substrate for the transfer machinery and to suppress T-DNA transfer to plants (Binns et al. 1995). Remarkably, the T-DNA transfer system also conjugatively transfers plasmid substrates to other agrobacterial cells (Beijersbergen et al. 1992), the yeast *Saccharomcyes cerevisiae* (Bundock et al. 1995, Piers et al. 1996) and other fungal cells (de Groot et al. 1998, Bundock et al. 1999, Gouka et al. 1999) and even human HeLa cells (Kunik et al. 2001). In this regard, the T-DNA transfer system is the most versatile of all conjugation systems known to function in bacteria.

Two other sets of observations further broadened the scope of conjugation functionality and ultimately led to naming of the T4SS superfamily. First, studies of plasmid transfer systems and the *A. tumefaciens* system in the 1980’s supplied evidence for the transfer of proteins to other bacteria or plant cells (Otten et al. 1984, Merryweather et al. 1986, Christie et al. 1988, Rees and Wilkins 1989). Second, sequence comparisons led to the discovery that homologs of the plasmid-encoded MPF proteins and the VirB proteins were also encoded within the *Bordetella pertussis ptl* gene cluster, whose products mediate secretion of the multisubunit Pertussis Toxin (PT) from the periplasm to the extracellular milieu (Shirasu and Kado 1993, Weiss et al. 1993, Winans et al. 1996). These remarkable findings established that conjugation machines are not only dedicated DNA translocation systems but also have evolved to deliver proteins across the cell envelope. In 1994, G. Salmond noted the conservation of VirB subunits among the *A. tumefaciens* T-DNA transfer and *B. pertussis* Ptl systems (Salmond 1994). He raised the interesting question, still under study today, of how a conserved VirB apparatus could access distinct types of substrates—oncogenic T-DNA and multisubunit PT—from different cellular compartments and then translocate these substrates to different destinations—plant cells or the extracellular milieu. He proposed the type IV nomenclature for these two VirB machines to distinguish them from the previously described I, II, and III secretion systems. In 1997, P.J. Christie broadened the T4SS designation to include not only the *A. tumefaciens* VirB and *B. pertussis* Ptl systems, but also the bacterial conjugation systems and the *H. pylori* Cag system (Christie 1997). In the ensuing years, the *Legionella pneumophila* Dot/Icm and many other effector translocator systems functioning in Gram-negative bacteria, all conjugation systems including those encoded by plasmids, conjugative transposons, or integrative and conjugative elements (ICEs), and more recently described interbacterial toxin delivery systems were added to this list (Cascales and Christie 2003, Bhatty et al. 2013, Grohmann et al. 2018, Costa et al. 2023, Breidenstein et al. 2025). With such diverse activities, the T4SSs are collectively the largest and most functionally versatile of the known bacterial secretion systems.

## A proposed nomenclature for the T4SSs and relaxosomal components

When MGEs were first discovered and characterized, the associated transfer genes characteristically were assigned MGE-specific, four-letter identifiers that adhered to the mnemonic proposed by Demerec et al. (1966), e.g. F plasmid-encoded *traA, traB, traC*, R388 plasmid-encoded *trwA, trwB, trwC*, RP4 plasmid-encoded *trbA, trbB, trbC*. Although this nomenclature is convenient for studies of a given conjugation system, no uniform nomenclature exists to facilitate comparisons of different MGE-encoded systems. This can be attributed to the fact that the transfer gene clusters have undergone extensive recombinogenic shuffling, gene acquisitions, and gene losses during evolution. Accordingly, similarly named genes often encode proteins with completely different functions, e.g. F plasmid *traA*, R388 *trwA*, and RP4 *trbA*, respectively, code for a pilin subunit, a Dtr auxiliary factor, and a transcription regulator. The discovery of effector translocators and T4SSs with other functions resulted in additional system-specific nomenclatures, further complicating efforts to compare components of the various systems. Partly because the *A. tumefaciens* VirB/VirD4 system emerged early on as a paradigm for the T4SS superfamily and partly due to the unusual naming system adopted for its components, the VirB1-11/VirD4 designations can serve as a convenient unifying nomenclature for the shared core components of T4SSs. For all *virB*/VirB-like genes/subunits associated with T4SSs, we thus propose that the field adopts a nomenclature in which the original name of the gene/subunit is followed with a subscript denoting the relevant *virB*/VirB gene/subunit, e.g. F-encoded TraA_B2_, TraL_B3_, TraC_B4_. In various T4SSs, the same functional genes exist in 2 or more copies. In such cases, we propose maintaining this nomenclature and adding a subscript numeral to distinguish the copies. The numeral assignment should respect the alphabetic order of the corresponding gene designation. Accordingly, in the pCF10 conjugation system, the two encoded VirB8 homologs are designated PrgD_B8-1_ and PrgL_B8-2_, whereas in the pIP501 system, the two VirB8 homologs are TraH_B8-1_ and TraM_B8-2_.

We further propose “D4” subscript nomenclature for all T4CP substrate receptors of T4SSs. It turns out that all T4CPs are members of the SpoIIIE/FtsK/HerA ATPase superfamily, yet they exhibit extensive variability in sequences, phylogenies, and known or predicted structures (see below) (Guglielmini et al. 2013). The D4 nomenclature is thus intended as a generic representation of this large subfamily of the SpoIIIE/FtsK/HerA ATPases whose common functions are to recruit substrates to T4SS channels. As mentioned above, there is increasing evidence that VirD4-like T4CPs act in concert with other factors to recruit substrates to cognate T4SSs. As best illustrated with the *L. pneumophila* Dot/Icm system, DotL_D4_ assembles together with DotM, DotN, DotY, and DotZ to form the T4CC, which functions to orchestrate the delivery of hundreds of effectors to the eukaryotic host during *L. pneumophila* infection (Kwak et al. 2017, Kim et al. 2020, Meir et al. 2020). As other examples, in the F plasmid conjugation system, TraD_D4_ forms specific contacts with the TraM Dtr factor as a prerequisite for efficient recruitment of the F plasmid substrate to the F-encoded T4SS (Wong et al. 2011, Wong et al. 2012). In the *Helicobacter pylori* Cag system, the chaperone CagF stably docks with Cagβ_D4_ for recruitment of the CagA substrate to the Cag T4SS (Couturier et al. 2006, Pattis et al. 2007, Bonsor et al. 2013). These findings support a general proposal that T4CPs assemble with other auxiliary proteins as T4CCs to recruit and spatiotemporally orchestrate the delivery of substrates into cognate T4SSs (Meir et al. 2018, Meir et al. 2020, Macé et al. 2022). Accordingly, we propose that proteins shown to interact with T4CPs to yield functional T4CCs be identified with the subscript D4Aux, e.g. for the Dot/Icm system, DotM_D4Aux_, DotN_D4Aux_, etc.

In Table 1, we list representative T4SSs discussed in this review, and names of their core VirB/D4 components along with various distinguishing features.

**Table 1.**
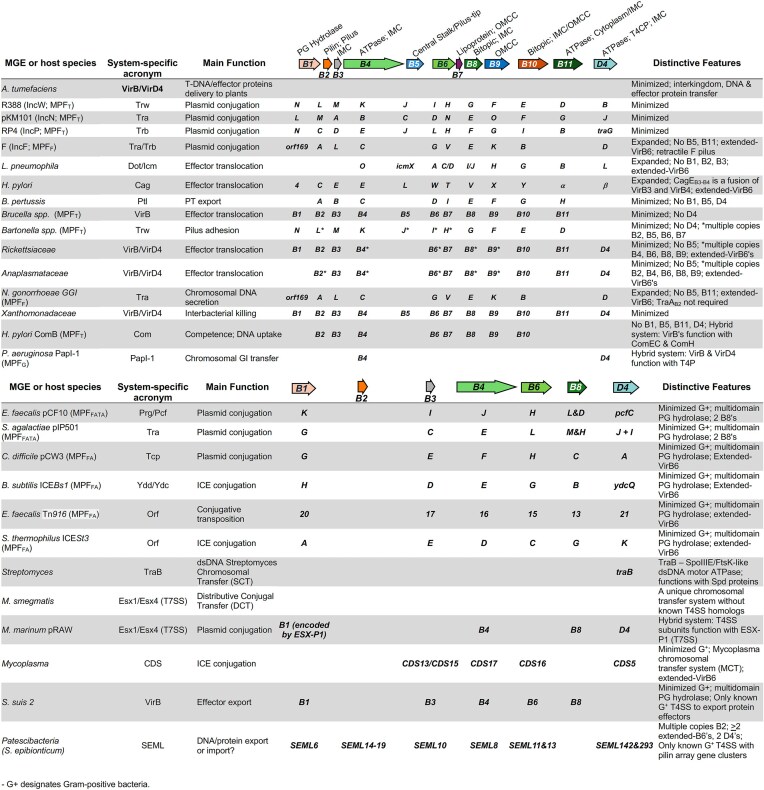
Overview of protein names in well-known T4SSs from Gram-negative and Gram-positive bacteria.

The same nomenclature problems arose with the Dtr proteins, namely, similar letters or numbers were assigned to Dtr proteins with completely different functions across the different MGEs. We therefore propose adding “Rel” or “Aux” as a subscript to indicate that the gene/protein corresponds to the relaxase or to an auxiliary Dtr protein, respectively. For example, TrwC_Rel_, MbeA_Rel_, and Orf20_Rel_ specify the relaxases, respectively, encoded by R388 plasmid, ColE1 plasmid, and the ICE Tn*916*, while TrwA_Aux_, MbeC_Aux_, and Orf22_Aux_ specify their cognate auxiliary factors (see below the Relaxosome section). Although conjugation systems encode only one relaxase, they can encode more than one auxiliary protein; in such cases, we propose adding a subscript numeral to distinguish the proteins by respecting the alphabetic order of the corresponding gene designation, e.g. F plasmid TraJ_Aux-1_, TraM_Aux-2_. Additionally, in some cases, different conjugation systems have appropriated the same 4-letter key for the relaxase, e.g. TraI_Rel_ for the F and RP4 transfer systems, despite extensive sequence and structural variations. If required to clarify discussions within a single paper, we propose adding the name of the MGE after the subscript denoting the relevant relaxase, e.g. TraI_Rel-F_, TraI_Rel-pKM101_. Similarly, for cross system comparisons, the MGE name is added as a subscript to redundantly named VirB or VirD4 proteins, e.g. TraF_B10-pKM101_ and TraB_B10-F_ or TraD_D4-F_ and PcfC_D4-pCF10_.

In Table 2, we list relaxosome components associated with representative conjugation systems from Gram-negative and Gram-positive bacteria, their phylogenetically-based affiliation, and short descriptions of subunit characteristics/functions.

**Table 2.** Main relaxase models associated with their encoding MGE, relaxase family, and corresponding 3D fold.

MGE	Host species	Relaxase name	Relaxase family	Specificities	Fold/relationship	PDB#
R388	*E. coli*	TrwC	MOB_F_	2Y in motif I	HUH	1OMH (Guasch *et a l*. 2003)
F	*E. coli*	TraI	MOB_F_	2Y in motif I	HUH	1P4D (Datta et al. 2003)
R1	*E. coli*	TraI	MOB_F_	2Y in motif I	HUH	5N8O (Ilangovan et al. 2017)
pCU1	*E. coli*	TraI	MOB_F_	2Y in morif I	HUH	3L57 (Nash *et al*. 2010)
RP4	*E. coli*	TraI	MOB_P_		HUH	
R64	*E. coli*	NikB	MOB_P_		HUH	
pTiC58	*A. tumefaciens*	VirD2	MOB_P_		HUH	
ColE1	*E. coli*	MbeA	MOB_P(HEN)_	HEN in motif III	HUH	
pCF10	*E. faecalis*	PcfG	MOB_P_		HUH	
RSF1010	*E. coli*	MobA	MOB_Q_		HUH	
R1162	*P. aeruginosa*	MobA	MOB_Q_		HUH	2NS6 (Monzingo et al. 2007)
pTiC58	*A. tumefaciens*	TraA	MOB_Q_		HUH	
pIP501	*E. faecalis*	TraA	MOB_Q_		HUH	
pLW1043	*S. aureus*	NES	MOB_Q_		HUH	4HT4 (Edwards *et al*. 2013)
pMV158	*Streptococcus agalactiae*	MobM	MOB_V_	Catalytic H	HUH	4LVI (Pluta et al. 2017)
pAD1	*E. faecalis*	TraX	MOB_C_		Restriction endonucleases	
CloDF13	*Enterobacter cloacae*	MobC	MOB_C_		Restriction endonucleases	
GGI	*N. gonorrhoeae*	TraI	MOB_H_	3H + HD motifs, 2 divalent cations	HD-hydrolases	
SXT/R391	*Vibrio cholerae/Proteus rettgeri*	TraI	MOB_H_	3H + HD motifs, 2 divalent cations?	HD-hydrolases	
Tn*916*	*E. faecalis*	Orf20	MOB_T_		*Rep_trans*	
ICE*Bs1*	*B. subtilis*	NicK	MOB_T_		*Rep_trans*	
ICE*St3*	*S. thermophilus*	RelSt3	MOB_T_		*Rep_trans*	
pCW3	*C. perfringens*	TcpM	MOB_M_		Tyr recombinases	

Y, H, E, and N indicate tyrosine, histidine, glutamic acid, and asparagine residues, respectively.

## Structural biology of Gram-negative T4SSs

**Figure 1.**
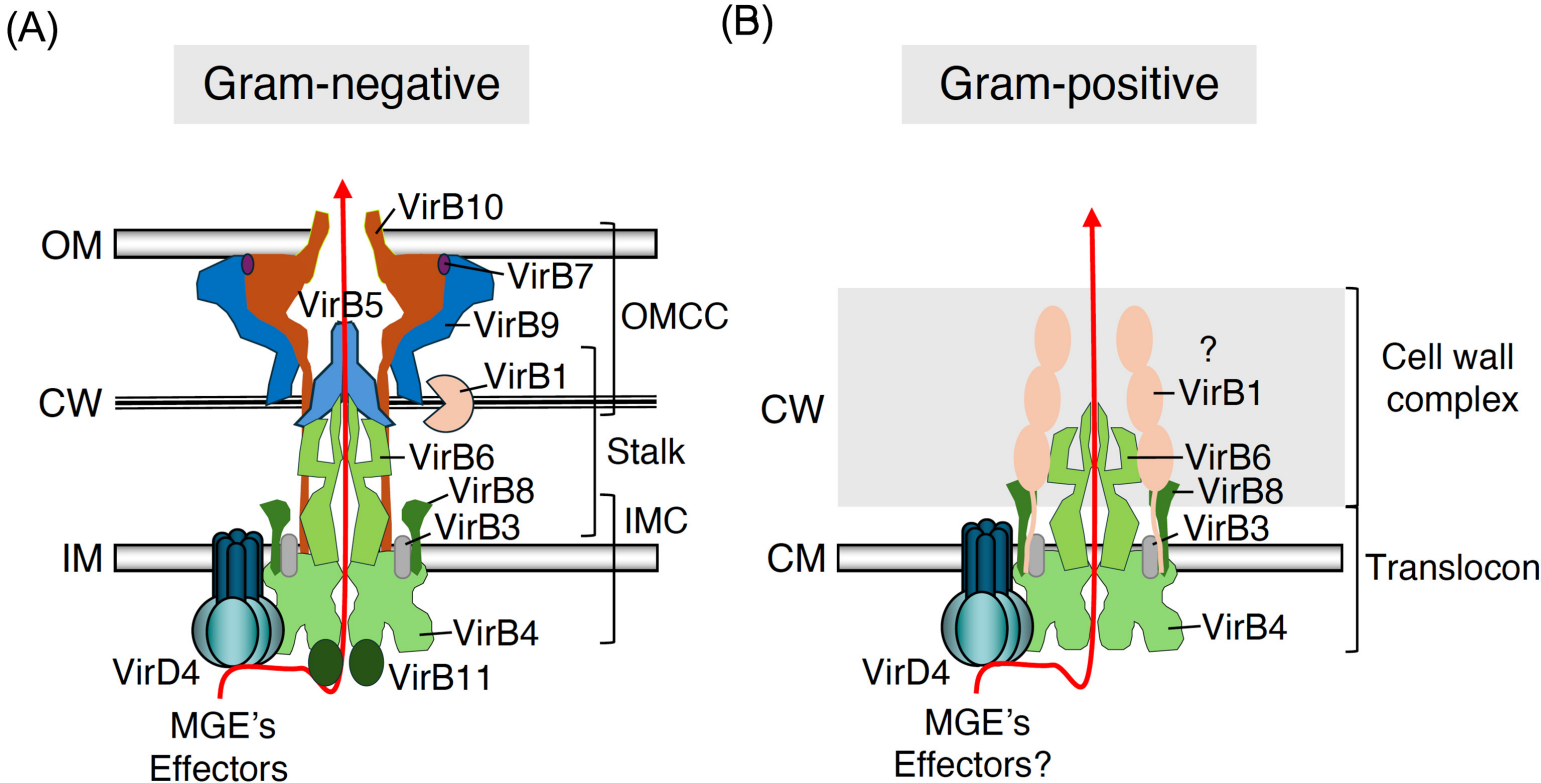
Representative models of T4SS in Gram-negative (A) and Gram-positive (B) bacteria. Subunit components and their relative positions in the T4SSs are indicated. The proposed translocation pathways for MGE’s or effector proteins are shown by lines with arrows. The (?) indicates incertanity regarding the positioning of VirB1 and VirB8 subunits relative to the cell wall width. OMCC: outer membrane core complex, IMC: inner membrane complex, OM: outer membrane, CW: cell wall, IM: inner membrane, CM: cytoplasmic membrane.

**Figure 2.**
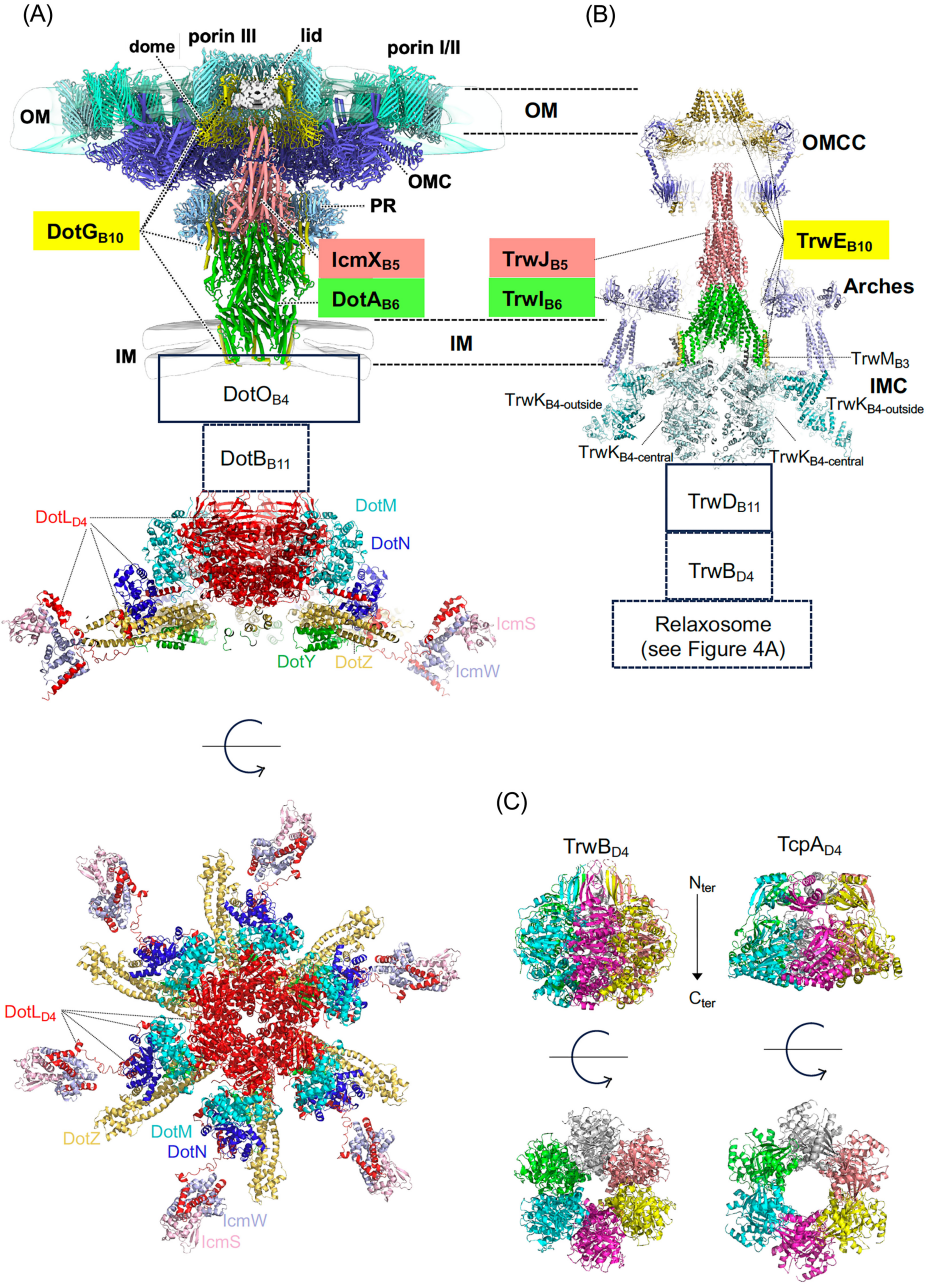
Near-atomic resolution structures of large T4SS-related subcomplexes. (A) The *Legionella* Dot/Icm T4SS structure (top) (Yue et al. 2025). The corresponding T4CC composed of the DotL_D4_ T4CP and auxiliary proteins docked onto the C-terminal domain of DotL_D4_ is shown below the Dot/Icm structure, in side and end-on views (Meir et al. 2020). (B) The conjugative R388 T4SS structure (Macé et al. 2022, Macé and Waksman 2024). Components of the Dot/Icm and R388 structures are labeled. Boxes with solid black lines indicate components of T4SS for which the location has been ascertained, but not their high-resolution structures. Boxes with dotted lines indicate components for which the high-resolution structure is known, but their location within the system is not yet known at high-resolution. The structure of TrwC_D4_ is not shown as it has not been determined yet in a complex with other proteins. (C) Side and top views of the X-ray structures of TrwB_D4_ (TrwB_D4_ΔN70, 1E9S) (Gomis-Ruth et al. 2001) and TcpA_D4_ (TcpA_102-530_, 6OZ1) (Traore et al. 2023) lacking their N-terminal transmembrane domains. We thank Yue J. for the top portion of Panel A depicting the Dot/Icm T4SS structure.

The 11 VirB proteins are core subunits required for full functionality of most T4SSs in Gram-negative bacteria (Fig. 1A and Table 1). Three ATPases, VirD4, VirB4, and VirB11, are required to energize machine assembly and substrate transfer, whereas only VirB4 and VirB11 are required for elaboration of conjugative pili. All three ATPases belong to the AAA + ATPAse superfamily (Guglielmini et al. 2013). They assemble as homohexamers, although each hexamer is structurally distinct. As mentioned above, VirD4 T4CPs are ancestrally related to the SpoIIIE/FtsK/HerA ATPase superfamily of dsDNA motor proteins (Gomis-Ruth et al. 2001). Available structures for R388-encoded TrwB_D4_ (Gomis-Ruth et al. 2001), pCW3-encoded TcpA_D4_ (Traore et al. 2023), and *L. pneumophila* DotL_D4_ (Kwak et al. 2017, Meir et al. 2020) further indicate that the T4CPs adopt hexameric structures similar to the dsDNA motors. Although some T4SSs lack VirD4 T4CPs, the VirB4 ATPases are signature subunits of all T4SSs described to date. This is intriguing because, like the VirD4 ATPases, the VirB4 ATPases also group phylogenetically with the SpoIIIE/FtsK/HerA ATPases (Guglielmini et al. 2013) yet have not been shown to function directly in substrate recruitment, although interactions with DNA substrates have been reported (Durand et al. 2010, Li et al. 2012). The VirB4 ATPases also adopt architectures distinct from the VirD4 T4CPs (Fig. 2B, lower panel and Fig. 2C). They assemble as six dimers arranged so that one protomer of the dimers forms an inner ring and the other forms an outer ring; the two rings surround the entrance to the central channel through which substrates are envisioned to pass (Hu et al. 2019, Hu et al. 2019, Macé et al. 2022). The protomers of each dimer are connected by their N-terminal domains (NTDs), which also embed into the inner membrane. Additionally, the NTDs of protomers comprising the inner and outer hexameric rings form specific contacts with the VirB3 and VirB8 subunits, respectively (Macé et al. 2022, Macé and Waksman 2024, Waksman 2025). This interaction network anchors the VirB4 hexamer of dimers in the inner membrane and potentially enables ATP-dependent structural changes. Although hexamer of dimers is the most commonly observed assembly for VirB4 subunits, another assembly mode was initially observed by Low et al. (2014), where the 12 VirB4 subunits form side-by-side barrel-like trimers of dimers. The functional significance of the latter assembly mode remains to be determined. However, because subsequent studies identified a VirD4 hexamer between the two barrel-like VirB4 trimers of dimers in the R388 T4SS structure, this assembly mode has been hypothesized to prevail in the DNA-conjugating architecture of the system (Waksman 2019, Waksman 2025).

The VirB11 ATPases assemble as double-stacked hexamers, whereby the N- and C-domains form the upper and lower hexameric rings, respectively (Yeo et al. 2000, Savvides et al. 2003, Hare et al. 2006, Prevost and Waksman 2018). Intriguingly, these ATPases appear to associate dynamically with the T4SS channel by forming ATP-dependent interactions with the inner rings of the VirB4 hexamers (Chetrit et al. 2018, Park et al. 2020, Macé et al. 2022). VirB11 ATPases are required for function of most T4SSs in Gram-negative bacteria, although curiously, not the F plasmid-encoded T4SS. These ATPases are also not associated with T4SSs found in Gram-positive bacteria.

The three ATPases interact to coordinate machine assembly and early-stage substrate processing reactions at the base of the T4SS (Cascales and Christie 2003, Atmakuri et al. 2004). One or more of the ATPases bind components of the large inner membrane complex (IMC), which consists of polytopic subunits VirB3 and VirB6, and bitopic subunits VirB8 and VirB10 (Macé et al. 2022, Macé and Waksman 2024, Waksman 2025). As discussed further below, a high-resolution structure of R388-encoded Trw system has unveiled striking details concerning the IMC architecture and subunit stoichiometries (Macé et al. 2022, Macé and Waksman 2024, Waksman 2025). Most intriguingly, the VirB6 subunit has long been proposed to assemble as a multi-membrane-spanning component of the IM channel, reminiscent of SecY of the SecYEG translocon. Remarkably, the R388 structure showed instead that the bulk of the VirB6 subunits assemble as a pentameric central structure in the periplasm and that only two N-proximal α-helices anchor the VirB6 subunits in the IM (Macé et al. 2022 and below for further details). VirB6 subunits are highly conserved among all T4SSs in Gram-negative and -positive bacteria, although they can range in size from that of *A. tumefaciens* VirB6, which is composed mainly of 5–6 hydrophobic α-helices, to considerably larger (Alvarez-Martinez and Christie 2009). These latter, so-called extended-VirB6’s characteristically have 5 or more hydrophobic α-helices but additionally one or more central or C-proximal large hydrophilic domains. How the more complex domain architectures of the extended VirB6’s contribute to the structures or functions of associated T4SSs is not yet known.

Two VirB subunits, VirB2 and VirB5, are required for substrate transfer through many T4SSs functioning in Gram-negative bacteria. With one recently identified exception of T4SSs required for survival of Patescibacteria (see below), these VirB subunits are not found among T4SSs functioning in Gram-positive bacteria. VirB2 is a pilin subunit that assembles as the extracellular conjugative pilus (Costa et al. 2016). Conjugative pili function mainly as attachment organelles to promote mating pair formation, although the F pilus has also been shown to mediate the passage of DNA substrates (Babic et al. 2008, Goldlust et al. 2023, Beltran et al. 2024). VirB5 assembles on the pilus tip (Aly and Baron 2007), and recent structural findings have led to a model in which a VirB5 pentamer initiates pilus assembly by docking on top of the VirB6 pentameric platform (Macé et al. 2022). VirB6 then recruits the VirB2 pilin subunits to the base of VirB5 in a reiterative process that results in growth of the pilus fiber from its base and extrusion of the VirB5 tip complex across the OM and extracellularly. VirB2 pilins appear to be required for function of most T4SSs in Gram-negative species, regardless of whether these systems elaborate conjugative pili (Jakubowski et al. 2005). Curiously, however, VirB5 subunits are absent from a number of Gram-negative T4SSs. This includes the F plasmid system, which is known to elaborate dynamically extending and retracting F pili, as well as other T4SSs not thought to elaborate pilus structures, e.g. *B. pertussis* Ptl, *N. gonorrhoeae* GGI, *H. pylori* ComB (Hamilton et al. 2005, Alvarez-Martinez and Christie 2009, Fernandez-Gonzalez and Backert 2014).

Three VirB subunits, VirB7, VirB9, and VirB10, assemble as the OMCC, which is a large substructure associated with all T4SSs functioning in Gram-negative species (Costa et al. 2023). The main functions of the OMCC are to mediate passage of substrates across the OM and, when present, direct formation of conjugative pili. As Gram-positive bacteria lack OM’s and also do not elaborate conjugative pili, their T4SSs do not carry the VirB subunits required to build OMCCs. The OMCCs are intrinsically stable substructures and, consequently, several OMCC structures associated with minimized and expanded T4SSs have been solved (Costa et al. 2021, Costa et al. 2023). Invariably, these are large, ≥3 MDa structures typically composed of 14 or more copies of each of the VirB subunits. The OMCCs characteristically possess inner (I) layer (also termed the Periplasmic Ring or PR) and an outer (O) layer. The I-layer is dominated by multiple copies of VirB9 N-terminal domains. The O-layer consists of an outer ring, which is dominated by multiple copies of VirB9 C-terminal domains and the VirB7 lipoprotein, and an inner ring dominated by multiple copies of VirB10 C-terminal β-barrel domains. OMCCs associated with minimized systems are built only from these VirB homologs, whereas those of expanded systems have appropriated additional subunits of unknown ancestries and are considerably larger. Structural details of various OMCCs have been reviewed recently (Costa et al. 2021, Sheedlo et al. 2022). Here, we will focus instead on summarizing recent advances in the structural definition at near-atomic resolution of the *L. pneumophila* Dot/Icm and plasmid R388-encoded T4SSs. These studies have identified several intriguing and novel features of OMCCs as well as other substructures of these nanomachines.

Recent breakthroughs in the elucidation of the structures of large T4SS complexes at near-atomic resolution have provided crucial mechanistic insights into (i) effector transfer by the expanded *Legionella* T4SS system (Yue et al. 2025) and (ii) pilus biogenesis and DNA transfer for conjugative T4SS systems (Macé et al. 2022, Macé and Waksman 2024) and the relaxosome (Williams et al. 2025).

In Legionella’s Dot/Icm T4SS, the OMCC exhibits three layers characterized by symmetry mismatches: a dome-shaped layer with 16-fold symmetry, a 13-fold symmetric outer membrane cap (OMC), and an 18-fold symmetric periplasmic ring (PR). The OMCC is composed of DotC_B7-1_, DotD_B7-2_, DotF, DotG_B10_ (C-terminal), DotH_B9_, and DotK, along with three species-specific components, Dis1, Dis2, and Dis3 (Durie et al. 2020, Sheedlo et al. 2021). Using *in situ* cryo-EM and cryo-ET, Yue and colleagues (Yue et al. 2025) recently provided remarkable views of the protein environment surrounding the OMCC and also of the central part of the IMC. They discovered a network of three porin rings organized around the OMCC’s outer-membrane channel (labeled porin I to III in Fig. 2A, top panel), with the porin I ring stabilized by another species-specific component, Dis4, while the porin III ring “hugs” the outer-membrane channel and dome, which they firmly established is made of DotG_B10_. Much has been written on distinct and common features of T4SS OMCCs that will not be repeated here (Costa et al. 2023). However, in light of the fact that porins have co-purified with R388 minimized T4SS (Low et al. 2014), it is tempting to suggest that the presence of arrays of porins surrounding the OMCC as seen by Yue et al. in the expanded *Legionella* system might be a general feature of T4SSs (Yue et al. 2025). The functional role of these porin arrays remains, however, to be determined. Also, the new higher resolution structure of the *Legionella* OMCC provides insights on DotG_B10_ that add to the set of common features this protein has with VirB10 homologues of the minimized conjugative systems (such as TrwE_B10_, recently observed by Macé and Waksman 2024): indeed, now in both R388 and Dot/Icm, most parts of the VirB10 homologue (TrwE_B10_ and DotG_B10_, respectively) can be traced and models built for these parts, confirming the central role that this protein plays in T4SS structure and biology (Fig. 2A, top panel and Fig. 2B).

The near-atomic cryo-EM structure of the Dot/Icm T4SS also reveals that two proteins essential for effector translocation, DotA_B6_ and IcmX_B5_, form a pentameric assembly in the central axis of the intact Dot/Icm machine (Yue et al. 2025). Cryo-ET and subtomogram averaging further capture the activated state of the secretion machine, demonstrating that a major conformational change in the DotA-IcmX protochannel is required to form an extended trans-envelope channel and to enable direct translocation of substrates from the bacterial cytoplasm into recipient cells. The authors point to the striking similarities in oligomerization state between DotA_B6_/IcmX_B5_ in TrwI_B6_/TrwJ_B5_. Like DotA_B6_/IcmX_B5_ in *Legionella*, TrwI_B6_/TrwJ_B5_ are centrally positioned and are pentamers. In both systems, the VirB6/VirB5 complex is proposed to conform to a portion of the translocation channel. In the R388 system, however, this complex is additionally postulated to act as a platform for pilus assembly, enabling the TrwL_B2_ pilin to dock and initiate pilus nucleation on top of TrwI_B6_ (reviewed in Waksman 2025). However, one must caution that the model proposed in Macé et al. (2022) remains to be tested; moreover, even if it turns out the TrwI_B6_ is indeed the platform on top of which the pilus is built, this protein would still block DNA transfer as, in the structure of the R388 T4SS system, the TrwI_B6_ pentamer, although centrally located, does not form a channel. Since DNA transfer can occur through the pilus (Goldlust et al. 2023, Beltran et al. 2024), DNA must also pass through TrwI_B6_ before inserting into the pilus lumen, which implies that it might also form a protochannel that awaits contact of the pilus with recipient cells during conjugation to open up into a channel, the same way DotA opens up when contact with a eukaryotic cell is made. It is worth noting that AlphaFold3 models VirB6 homologs in various open states with high confidence, indicating that such an opening is supported by the VirB6 pentameric complexes (Breidenstein et al. 2025).

Previous cryo-ET work on the Dot/Icm system revealed a hexamer of dimer architecture for the T4SS IMC, whereby DotO_B4_ is present in 12 copies, six of which form a central hexamer as expected from a AAA + ATPase and six additional copies form dimers with each of the central DotO_B4_ subunits. The near-atomic structure of this part of the IMC remains to be determined for the *Legionella* system but progress towards this goal was achieved with the IMC of the R388 system (Macé et al. 2022, Macé and Waksman 2024). The IMC in the R388 T4SS is made of 24 N-terminal trans-membrane tails of TrwG_B8_, six copies of TrwM_B3_, and 12 copies of TrwK_B4_. The IMC therefore results from the hexamerization of a protomer containing four N-terminal tails of TrwG_B8_, one copy of TrwM_B3_, and two copies of TrwK_B4_, with one TrwK_B4-central_ assembling in the hexamer to form the central ATPase hexamer referred to above and the other, TrwK_B4-outside_, forming with each TrwK_B4-central_ subunit a dimer (Fig. 2B). It is unclear why six additional copies of VirB4 homologues are required for function when a central hexameric entity should be sufficient. So far, the only functional difference between VirB4-central and VirB4-outside is that TrwK_B4-central_ uniquely interacts with TrwM_B3_, while TrwK_B4-outside_ subunits each interact with 4 N-terminal tails of TrwG_B8_. Thus, the VirB4-outside subunits in the protomer appear to play a structural role in supporting the TrwG_B8_ arches, while the VirB4-central subunit functions structurally by supporting TrwM_B3_ and enzymatically by functioning as a motor ATPase.

The VirB11 homologues of both R388 (TrwD_B11_) and Dot/Icm (DotB_B11_) T4SSs have been located just under the VirB4-central hexamer (Macé et al. 2022, Yue et al. 2025). However, how these two ATPases may work together in concert is unknown. Finally, it remains unclear where the VirD4-like substrate receptor or larger T4CC locates and how it interacts with the machinery. The only specific interaction that has been characterized between the T4CP and the conjugative R388 T4SS is through the N-terminal cytoplasmic end of TrwE_B10_ (Llosa et al. 2003), but no molecular details have been obtained. As mentioned above, in *Legionella*, DotL_D4_ assembles together with DotM_D4Aux_, DotN_D4Aux_, DotY_D4Aux_, and DotZ_D4Aux_ as well as the chaperone module IcmS/W to build the T4CC (Meir et al. 2020; Fig. 2A, bottom panel). The latter proteins all interact with DotL_D4_ through its long C-terminal tail (Meir et al. 2020, Fig. 2A). In the conjugative minimized R388 system, the coupling protein, TrwB_D4_, also contains a C-terminal tail, but this tail is used to recruit the substrate (the relaxase-DNA nucleoprotein) directly through interaction with the auxiliary relaxosome component TrwA_Aux-R388_ (Llosa et al. 2003). In contrast, in *Legionella*, the sites of interactions of effectors with the T4CC are on IcmS/W and also DotM_D4Aux_ (Meir et al. 2018), not DotL_D4_.

### *In situ* structures

Structures of several T4SSs assembled in the native environment of the cell envelope have also been solved by *in situ* cryoelectron tomography. These structures were solved at resolutions considerably lower than the recently described R388 and Dot/Icm machine structures, but nevertheless add important details not observed with the *in vitro* structures. For the F plasmid-encoded T4SS, for example, structures were solved of the translocation channel presumptively in its inactive state (Hu et al. 2019). This structure is dominated by the central hexamer-of-dimer arrangement of the VirB4 homolog at the entrance to a channel that extends across the IM and through a central cylinder in the periplasm. A structure of the translocation channel bound to the F pilus also showed extensive conformational changes in the central cylinder and OMCC associated with F pilus biogenesis. Finally, and most remarkably, F pili were visualized in association with basal structures that are completely distinct from the characteristic channel complexes composed of large OMCC and IMC subassemblies. Rather, the basal platforms are configured as thin, mushroom-capped stalk structures extending across the periplasm and contacting the F pilus at the outer membrane, or simply as small densities at the outer membrane associated with the F pilus without an underlying periplasmic structure. These findings raise intriguing questions relating to the biogenesis of F pili and how assembled pili are deposited onto alternative basal platforms and for what biological ends. Other *in situ* structures of the pKM101, *H. pylori* Cag, and *L. pneumophila* Dot/Icm systems have also unveiled features of interest, most notably, the universally conserved hexamer-of-dimer arrangements of the VirB4 homologs, docking of the VirB11 hexamer at the base of VirB4’s central hexamer, and in the case of the Cag system peripheral densities surrounding the VirB4/VirB11 complex that are contributed by Cagβ_D4_ (Chetrit et al. 2018, Hu et al. 2019). Whereas pKM101’s periplasmic stalk assembled from VirB6 and VirB5 presents as a narrow structure devoid of a discernible channel, the equivalent structures associated with the F, Dot/Icm, and Cag systems are considerably larger and have well-defined central channels extending to the OMCC. In view of the high-resolution R388 T4SS structure and the recently described high-resolution *in situ* structure of the Dot/Icm machine, it is reasonable to predict that central stalks of the minimized systems as well as the central cylinders of the expanded T4SSs are similarly composed of homologs of VirB6 and VirB5 subunits.

## Structural and functional comparisons between Gram-negative and Gram-positive conjugative T4SSs

While several model systems of Gram-negative T4SSs are relatively well characterized in terms of structure and function, we know much less about their Gram-positive counterparts. The ones studied so far are conjugative T4SSs which, while demonstrating a strong capacity to facilitate horizontal DNA transfer, likely exhibit different architectures that are adapted to their cell envelope composition and structure. Comparative analyses of T4SS gene clusters from various conjugative Gram-positive elements have revealed considerable diversity in the composition of their transfer proteins. A commonly used classification is built on phylogenetic analysis of the conserved ATPase VirB4, which divides T4SSs into MPF classes. Gram-positive T4SSs fall into two major clades, the MPF_FA_ and MPF_FATA_ systems (Guglielmini et al. 2013). In both clades, most conjugation modules encode for a set of conserved proteins homologous to the Gram-negative VirB subunits. Many of the Gram-positive VirB homologs retain structural similarities with their Gram-negative counterparts despite very low (below 20%) sequence identity. Additionally, many systems also encode one or more system-specific proteins not present in Gram-negative systems.

A minimal T4SS model for Gram-positive bacteria was proposed by P. J. Christie (Fig. 1B, right panel) (Bhatty et al. 2013), suggesting that T4SSs assemble a channel that spans the entire envelope and consists of distinct functional modules. These include the translocon/ATPase complex that mediates DNA translocation across the cytoplasmic membrane—functionally analogous to the IMC in Gram-negative systems—and the cell wall complex that enables DNA passage through the thick peptidoglycan layer. Although this assembly model remains speculative, recent studies combining structural and functional analysis increasingly support its validity (Breidenstein et al. 2025, Maffo-Woulefack et al. 2025).

One unresolved question in the biology of T4SSs concerns the route of DNA translocation during conjugation. It is generally considered that the DNA will be guided through the OMCC and/or the pilus from the IMC to the recipient cell. However, the absence of the OMCC homologs in Gram-positive T4SSs raises questions about the mechanism by which the DNA crosses the thick PG-barrier to achieve efficient translocation. In these systems, several membrane proteins have been predicted or experimentally shown to contain large soluble domains that extend from the membrane to the extracellular environment, potentially contributing to this process.

### PG-degrading enzymes

**Figure 3.**
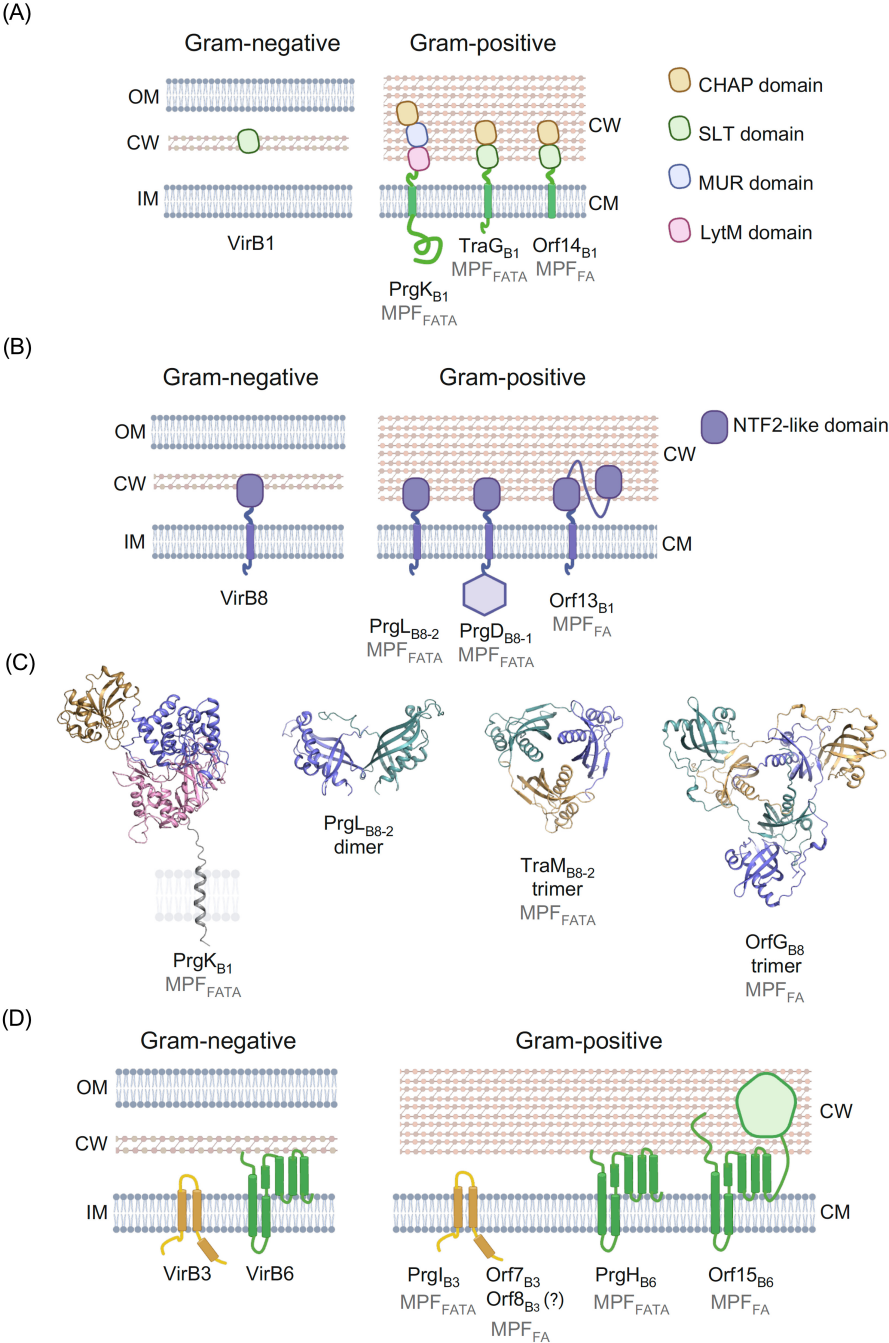
Comparative architectures and representative structures of cell wall complex subunits VirB1 and VirB8, and the translocon VirB3 and VirB6 homologs from Gram-negative and Gram-positive systems, including MPF_FATA_ and MPF_FA_ clades. (A) Schematic of VirB1-like PG-hydrolases from Gram-negative and Gram-positive systems; PrgK_B1_, TraG_B1_, and Orf14_B1_, respectively, from pCF10, pIP501, and Tn*916*. Domain annotations: CHAP (orange), SLT (green), MUR (blue), and LytM (pink). (B) Schematic of VirB8-like subunits from Gram-negative and Gram-positive systems, PrgL_B8-2_, and PrgD_B8-1_ from pCF10 and Orf13_B8_ from Tn*916*. NTF2-like domain (purple). (C) X-ray structures of PrgK_B1_ (8S0U) (Sun et al. 2024), PrgL_B8-2_ (7AED) (Jager et al. 2022), TraM_B8-2_ (4EC6) from pIP501 (Goessweiner-Mohr et al. 2013), and OrfG_B8_ (6PKW) from ICE*St3* (Maffo-Woulefack et al. 2025). (D) Schematic of VirB3 and VirB6 subunits from Gram-negative and Gram-positive systems; PrgI_B3_ from pCF10, and Orf7_B3_ and Orf14_B1_ from Tn*916*, PrgH_B6_ and Orf15_B6_ from pCF10 and Orf13_B8_ from Tn*916*. The topology of Orf15 remains uncertain, as the informatic analyses predict more transmembrane helices than those observed in the folded protein (Macé et al. 2022). OM: outer membrane, CW: cell wall, IM: inner membrane, CM: cytoplasmic membrane.

A main difference between Gram-negative and Gram-positive bacteria is the thickness of their cell wall. Gram-negative bacteria have a peptidoglycan (PG) layer in their periplasmic space. This layer is usually only a few nanometers thick, e.g. *E. coli*’s PG layer is ∼4 nm thick (Huang et al. 2008). Gram-positive bacteria have a much thicker PG layer, usually between 30 and 100 nm (Silhavy et al. 2010), which plays a critical function in maintaining the cellular integrity in these bacteria. Both in Gram-negative and Gram-positive systems, the PG layer is a barrier that needs to be crossed for conjugation to occur. In Gram-negative systems, the main player that modifies the PG layer is VirB1, which has a soluble lytic transglycosylase (SLT) activity (Llosa et al. 2000, Zupan et al. 2007). These soluble proteins are secreted to the periplasm (Fig. 3A, left panel). Once there, they locally disrupt the PG layer to allow for the formation of the MPF channel. However, while correct VirB1 function strongly facilitates T4SS biogenesis, it is not consistently essential for its function (Berger and Christie 1994, den Hartigh et al. 2004). In Gram-positive systems, the VirB1 homologs play similar roles. However, since they must act extracellularly, all known Gram-positive homologs are anchored in the membrane and have one or more extracellular enzymatic domains (Arends et al. 2013, Laverde Gomez et al. 2014) (Fig. 3A, right panel). It is not yet clear whether the disruption of the PG layer in Gram-positive systems is needed purely for the assembly of the channel, but it has been suggested that a local disruption of the PG layer around the T4SS is also needed to form a duct for the substrate to pass through during conjugation (Sun et al. 2024). VirB1 homologs have been classified into 4 classes, with the ALPHA, BETA, and DELTA classes found in Gram-positive systems, while the GAMMA class encompasses all known Gram-negative homologs (Fig. 3A) (Goessweiner-Mohr et al. 2014).

However, even within the same MPF class there are differences. PrgK_B1_ from pCF10 has 3 extracellular domains and thus belongs to the DELTA class (Laverde Gomez et al. 2014, Sun et al. 2024). Its middle domain has muramidase activity, not the standard SLT activity, and is the active PG-degrading domain. The other two domains of PrgK_B1_, LytM, and CHAP, seem to have a more regulatory function (Sun et al. 2024). However, other MPF_FATA_ examples belong to other classes, like the ALPHA class TraG_B1_ from pIP501 that has only 2 domains (SLT and CHAP) and a much shorter intracellular domain (Arends et al. 2013). The Orf14_B1_ protein from Tn*916*, a MPF_FA_ system, belongs to the BETA class. It is similar to the ALPHA class, but lacks an intracellular domain (Fig. 3A, right panel). It is clear that Gram-positive VirB1 homologs have adapted different enzymatic functionality to deal with the complex peptidoglycan in Gram-positive bacteria.

### The cell wall complex

VirB8 homologs from Gram-positive bacteria are consistently found in most conjugative systems (Goessweiner-Mohr et al. 2013). Over the past decade, several studies have focused on their structural and functional analysis (Porter et al. 2012, Goessweiner-Mohr et al. 2013, Kuroda et al. 2015, Fercher et al. 2016, Casu et al. 2018, Jager et al. 2022, Macé and Waksman 2024, Maffo-Woulefack et al. 2025). Collectively, these studies have underscored their importance for conjugative transfer, but intriguingly have highlighted the greater diversity of VirB8 homologs in Gram-positive bacteria in terms of topology and domain organization compared to their counterparts in Gram-negative systems. Despite this variability, they typically possess a single transmembrane domain and at least one NTF2-like domain—a hallmark shared with VirB8 from Gram-negative systems. Members of the VirB8 protein family have been categorized into three classes: ALPHA, BETA, and GAMMA, based on their domain organization (Goessweiner-Mohr et al. 2013). The ALPHA class comprises proteins with a single extracellular NTF2-like domain. The BETA class contains an additional NTF2-like domain, while the GAMMA class features an extra, uncharacterized domain predicted to be localized in the cytoplasm (Fig. 3B).

In MPF_FATA_, all known conjugative elements encode a VirB8 homolog from the ALPHA class. Some systems, such as those from pCF10 and pIP501, also encode an additional VirB8 homolog from the GAMMA class. For example, pCF10 encodes PrgD_B8-1_ and PrgL_B8-2_, which belong to GAMMA-class and ALPHA-class, respectively, (Goessweiner-Mohr et al. 2013, Jager et al. 2022) (Fig. 3B, right panel). Structural and biochemical analyses have shown that PrgL_B8-2_ assembles as dimers (Fig. 3C) and dodecamers in solution, while structural predictions using Alphafold3 suggest a decameric assembly as the most likely configuration. In contrast, structural modeling of PrgD_B8-1_ indicates a trimeric organization (Jager et al. 2022, Breidenstein et al. 2025).

In MPF_FA_ systems, particularly among elements from the Tn*916* superfamily of ICEs, only VirB8-like from BETA-class have been identified so far (Fig. 3B). Notably, TcpC_B8_ from pCW3, OrfG_B8_ from ICE*St3* and Orf13_B8_ from Tn*916* have been recently shown to assemble as trimers (Porter et al. 2012, Maffo-Woulefack et al. 2025; Fig. 3C), further supporting the idea that VirB8 homologs from the different classes act as multimers within their respective conjugative systems.

The precise contribution of VirB8 homologs in the assembly and architecture of the full T4SS channel is not fully known. In addition to a predicted critical role for T4SS structural integrity, VirB8 homologs have been shown to interact with VirB1 homologs (Porter et al. 2012, Kohler et al. 2017, Sun et al. 2024); and are therefore likely important for the proper localization of the VirB1 PG-degrading enzyme. Furthermore, an observed DNA-binding activity of OrfG_B8_ from ICE*St3* in *S. thermophilus* points to a potential role of VirB8 homologs in the formation of the DNA translocation channel through the cell wall (Maffo-Woulefack et al. 2025). Whether this function is conserved across other Gram-positive systems remains to be determined.

### The translocon

Most of the studied MPF_FATA_ and MPF_FA_ systems encode structural homologs of the IMC subunits including VirB3, VirB4, VirB6, VirB8, and the T4CP (Maffo-Woulefack et al. 2025). This conserved protein composition suggests that the Gram-positive T4SSs retain a similar DNA translocation mechanism as that deployed by the Gram-negative T4SSs across the cytoplasmic membrane. Interestingly, no structural homologs of VirB10 have been identified in Gram-positive systems. Although the bulk of VirB10 is incorporated into the OMCC, its N-terminal region spans the inner membrane. VirB10 is therefore the only known T4SS subunit that physically bridges the IMC to the OMCC in the Gram-negative cell envelope, a feature that appears to facilitate coordination of the entire T4SS in response to intracellular and extracellular signals (Cascales and Christie 2004, Cascales et al. 2013, Macé and Waksman 2024). The absence of VirB10 homologs in the Gram-positive systems suggests not only that these systems have adapted to the monoderm envelope architecture, but also that another unidentified component(s) of these T4SSs likely performs a VirB10-like function in relaying signals from the cell interior and exterior to activate the machines for transfer.

Except for VirB4 and T4CPs, which are easily identified using basic local alignment search tools (Gabler et al. 2020), the identification of other subunits of Gram-positive T4SSs is trickier due to the low sequence conservation and the absence of common domains. Consequently, additional topological and structural analyses are required.

The identification of VirB3 homologs is based on the known characteristics of VirB3, which are relatively small membrane proteins (10–15 kDa) containing two transmembrane domains, a C-terminal α-helix and short cytoplasmic extensions at both N- and C-termini (Fig. 3D). The genetic context of VirB3 homologs within T4SS clusters can aid their identification, as the *virB3* gene is typically located just next to *virB4*. This proximity, along with evidence of their co-evolution, further supports their functional association. In pCF10, PrgI shares several key features with known VirB3 proteins, such as a small size (117 residues) and two predicted transmembrane helices (Fig. 3D). *prgI_B3_* is flanked upstream by *prgH_B6_* and downstream, partially overlapping, with *prgJ_B4_*. Recent 3D modeling predicts that PrgI_B3_ binds to PrgJ_B4_ with a 1:1 stoichiometry, as observed for TrwM_B3_ and TrwK_B4_ from the R388 plasmid transfer system (Macé et al. 2022, Breidenstein et al. 2025). In the pIP501 *tra* cluster, *traC* and *traI* encode small proteins with two predicted transmembrane domains. TraI was proposed to anchor the coupling protein TraJ_D4_ to the cytoplasmic membrane. Like most other *virB3* genes, *traC* is located immediately adjacent to *traE_B4_*, supporting a proposal that *traC* encodes the VirB3 subunit for the pIP501 transfer system (Bhatty et al. 2013).

In MPF_FA_ systems, including Tn*916*, ICE*Bs1*, ICE*St3*, and pCW3, two proteins exhibit characteristics typical of VirB3 proteins (Maffo-Woulefack et al. 2025). Their genes are typically encoded upstream and adjacent to the *virB4* gene. The most well-characterized VirB3 homologs are TcpD and TcpE from pCW3 residing in *Clostridium perfringens* (*Wisniewski et al. 2015*). Both proteins are essential for the conjugative transfer of pCW3 and have been shown to localize in the cell envelope independently of other transfer proteins. *tcpE* resides immediately upstream of *tcpF_B4_*, and TcpE is more conserved across MPF_FA_ conjugative systems than TcpD, as evidenced by ConC from ICE*Bs1* and OrfE from ICE*St3*, which both belong to the TcpE family (pfam: PF12648). TcpE is therefore most likely the VirB3 homolog of the pCW3 transfer system.

Regarding the VirB6 homologs, their identification in both MPF_FA_ and MPF_FATA_ systems is straightforward. These proteins are integral membrane components characterized by having a predicted four to six transmembrane helices (Breidenstein et al. 2025)—a hallmark that distinguishes them from other transfer subunits (Fig. 3D). Recent comparative analyses and structural predictions indicate that VirB6 homologs from MPF_FATA_ systems closely resemble their Gram-negative counterparts, particularly in terms of length and predicted oligomerization as pentamers (Breidenstein et al. 2025) (Fig. 3D). Although homologs of VirB6 proteins from MPF_FA_ systems share these general features, they also exhibit distinctive characteristics reminiscent of the extended-VirB6 subfamily (Bao et al. 2009): they are notably larger (700–900 residues compared to around 300 residues for VirB6 proteins from MPF_FATA_) and contain additional unstructured domains (Fig. 3D).

### The energetic components

Unlike Gram-negative systems that often encode both VirB4 and VirB11 ATPases, Gram-positive systems universally lack VirB11 homologs. Despite their known importance in the conjugative process, detailed insights into the specific structural, biochemical, and functional characteristics of VirB4 homologs within the diverse T4SSs in Gram-positives remain misunderstood. Although they exhibit low sequence identity, these proteins retain all signatures motif of AAA + ATPases, including Walker A and Walker B motifs. The analysis of ATPase activity among different VirB4 homologs has yielded divergent results. For instance, PrgJ_B4_ from pCF10 and the VirB4 homolog from mCTn4 of *Clostridium difficile* have demonstrated ATPase activity, whereas ConE_B4_ from ICE*Bs1* was reported to be inactive (Li et al. 2012, Sorokina et al. 2021, Murthy et al. 2023). Regarding their structural assembly, biochemical analyses have shown that VirB4 proteins multimerize *in vitro*, with various oligomeric states: dimers for PrgJ_B4_, predominantly monomeric forms with some dimers and high-order complexes for ConE_B4_ and hexamers of VirB4 from CTn4 (Murthy et al. 2023). While DNA binding capacity has been clearly shown for several VirB4 proteins from Gram-negative T4SSs, only PrgJ_B4_ was reported to bind DNA *in vitro* and *in vivo*; these results suggest the direct involvement of VirB4 proteins in the DNA translocation during the conjugation process (Li et al. 2012).

Delivery of the DNA substrate to the secretion channel of Gram-positive conjugation systems is orchestrated by T4CPs, as is also the case for conjugation systems functioning in Gram-negative species (Atmakuri et al. 2003, Atmakuri et al. 2004, Redzej et al. 2017). Interestingly, for many conjugative systems, the T4CP-encoding gene is located in direct proximity to the relaxase-encoding gene, and furthermore, phylogenetic trees obtained for T4CPs and relaxases are often highly congruent (Garcillan-Barcia et al. 2009). Phylogenetic analyses have classified these proteins into two major families (Guglielmini et al. 2013): (i) the VirD4 family, including T4CPs from all Gram-negative T4SSs and Gram-positive MPF_FATA_ and MPF_FA_ systems, and (ii) the TcpA family represented by TcpA from pCW3 of *C. perfringens* as prototype, which is restricted to a subset of MPF_FA_ systems. As mentioned earlier, we propose appending the generic D4 subscript to all T4CPs to distinguish them from other members of the large FtsK/SpoIIIE/HerA ATPase superfamily. We acknowledge the T4CPs are highly diverse in sequence, phylogeny, structure, and possibly even functions as suggested by a recent study of pCW-encoded TcpA. This study showed that the TcpA hexamer more closely resembles FtsK than the structural archetype of the VirD4 family, R388-encoded TrwB_D4_ (Traore et al. 2023). These findings prompted the intriguing suggestion that the *C. perfringens* Tcp conjugation apparatus translocates its DNA substrate as a double-stranded intermediate instead of the ssDNA substrates translocated through the VirD4/TrwB-dependent systems.

### Adhesins vs pili

Gram-negative conjugation systems utilize conjugative pili to initiate mating pair formation, but Gram-positive systems do not code for conjugative pili. Instead, they often rely on cell-wall-anchored adhesin proteins to promote mating pair formation. Such proteins, mostly found in lactic acid bacteria, carry a C-terminal LPxTG motif that anchors them to the peptidoglycan in a sortase-dependent manner (Geoghegan and Foster 2017, Foster 2019). One of the best characterized examples is PrgB from pCF10 (Sun et al. 2023). The main functional domain of PrgB is the *polymer adhesin domain* or PAD. This domain is widely spread in Gram-positive bacteria, and the proteins are called Glucan-binding protein C, Dextran-binding lectin, or Antigen I/II in *Streptococcus*, and Aggregation substance in *Lactococcus* and *Enterococcus*. This domain is extensively reviewed in Jarva et al. (2020). Briefly, PrgB facilitates mating pair formation by binding to lipoteichoic acid (LTA), a major component of the Gram-positive peptidoglycan layer, of the recipient cell. This attachment anchors the donor and recipient cell together, strongly increasing the chances of a mating pair formation. This resembles the function of the F pili, which upon binding to the recipient cell retracts and thus pulls the recipient cell close to the donor cell.

Not all Gram-positive systems encode cell-wall-anchored surface adhesins with these polymer adhesin domains. The broad host range plasmid pIP501, for example, lacks such adhesins, which may contribute to its ability to transfer across diverse hosts. Recently, some systems have been proposed to use inactive VirB1 homologs as adhesins (Ortiz Charneco et al. 2024), which bind, but do not modify the peptidoglycan of the recipient cell. It is not yet established how widespread such a VirB1 adhesin function exists among the Gram-positive systems.

## The relaxosome of Gram-negative and Gram-positive systems

Bacterial conjugation is initiated by a key protein encoded by the conjugative MGEs, named relaxase, because of its ability to relax a supercoiled double-stranded DNA substrate. These proteins are transesterases that nick the strand of DNA (T-strand) to be transferred at a specific site within the origin-of-transfer (*oriT*) sequence (de la Cruz et al. 2010, Guzman-Herrador and Llosa 2019). The generated 3′OH extremity is used to initiate a round of rolling-circle replication. Relaxases are usually known to form a covalent adduct with the 5′P end of the cleaved DNA through its catalytic residue, which is usually a tyrosine residue. This relaxase-T-strand covalent adduct is thought to be transferred through the T4SS machinery to the recipient cell. Once in the recipient cell, the transesterase religates the 5′ and 3′ ends of the transferred ssDNA in a strand transfer reaction. The complementary strand is then synthesized by the DNA replication machinery of the host cell. To perform these DNA processing steps, relaxases usually act on two specific sites within the *oriT* sequence: a *bind* site that is first recognized, and a *nic* site that is cleaved after binding (Lucas et al. 2010). This two-step mechanism is thought to fit with most—if not all—relaxases studied up to now. Another major question was to know how the relaxase could pass through the conjugation pore as it would be too large in its natively folded form. This question was solved in 2018 when the TrwC_Rel-R388_ was fused to protein domains known to be resistant to unfolding, and these fusion proteins were no longer transferred to the recipient cell (Trokter and Waksman 2018). The relaxase is thus at least partly unfolded for transfer through the T4SS machinery. To catalyze nicking at cognate *oriT* sites, relaxases typically interact with one or more other factors called auxiliary proteins, These proteins usually interact with the *oriT* DNA and/or the relaxase, thus forming a nucleoprotein complex called the relaxosome (de la Cruz et al. 2010). Upon processing, the resulting relaxase-T-DNA complex is recruited to the T4SS channel through the action of the T4CP (Cabezon et al. 1997). The T4CP plays a central role in determining the T-DNA specificity by selectively recognizing the relaxosome complex destined for transfer (Hamilton et al. 2000). This specificity arises from physical interactions between the T4CP and the relaxasome (Szpirer et al. 2000, Schroder et al. 2002). In some systems, additional relaxosomal auxiliary factors also participate in the T4CP recognition, as demonstrated for the TrwA_Aux-R388_ and TraM_Aux-1-F_ (Disque-Kochem and Dreiseikelmann 1997, Llosa et al. 2003, Lu et al. 2008). Notably, although T4CPs interact specifically with relaxosomal proteins to specify substrate recognition, the nature of T4CP interactions with T4SSs is not yet defined. Curiously, there is evidence that a T4CP from a given conjugation system can productively engage with the T4SS machines of heterologous conjugation systems (Llosa et al. 2003).

In Gram-positive systems, although experimental evidence is limited, a similar mechanism of relaxosome-T4CP-mediated recruitment has been proposed. This is supported by the observed interactions between TraJ_D4_ and TraA_Rel_ and TraJ_D4_ and TraG_B1_ from pIP501, suggesting the direct involvement of T4CP in relaxosome recognition and recruitment to the T4SS channel (Abajy et al. 2007).

### Structure of a MOB_F_ relaxosome

**Figure 4.**
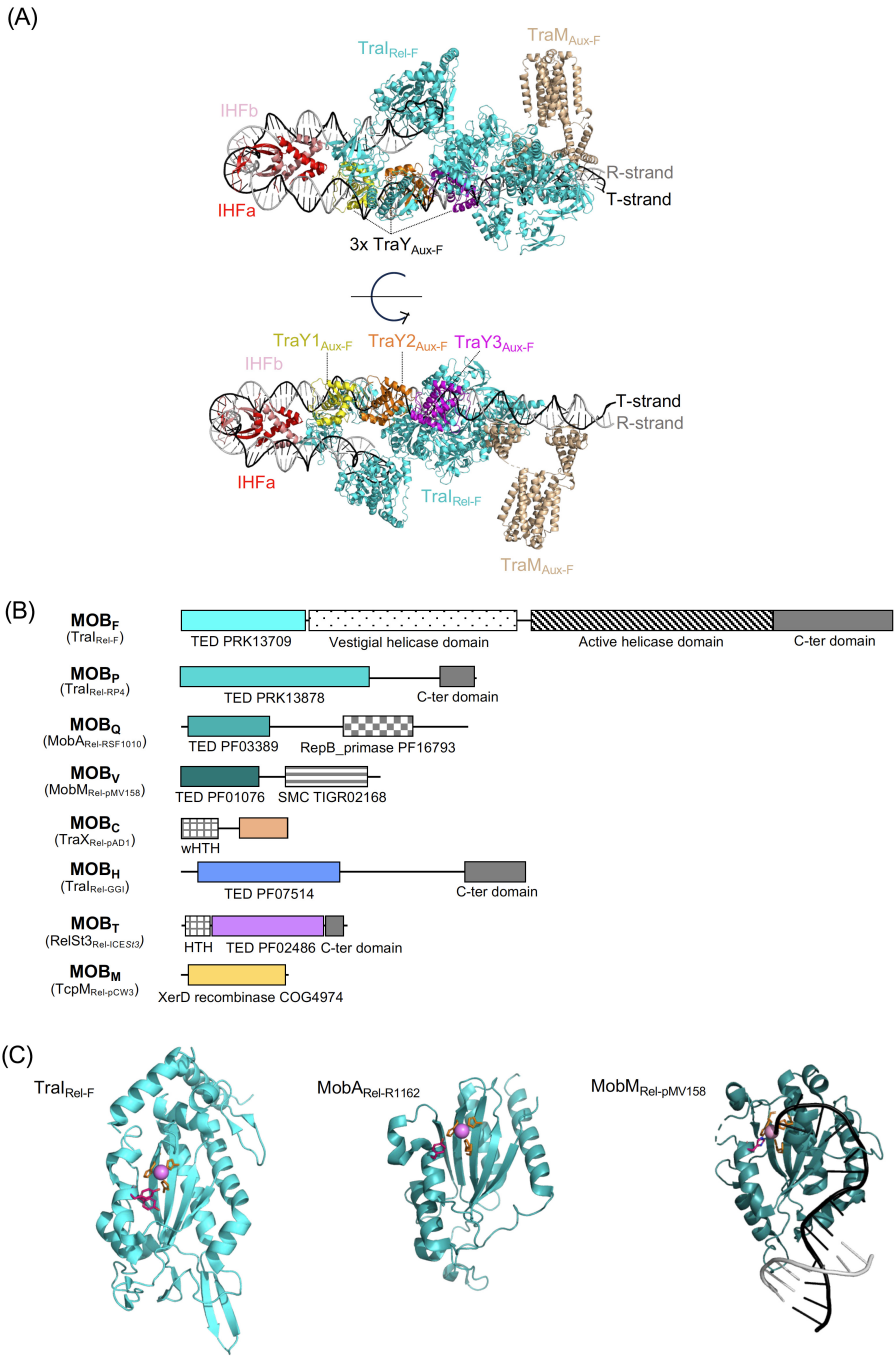
Structural organization of the relaxosome and diversity of relaxases. (A) Structure of the fully assembled relaxosome from the F plasmid (Williams et al. 2025). Proteins are labeled as are the DNA strands. (B) Schematic overview of representative relaxases from each family. The transesterase domain (TED) is shown in color, additional domains are in plain gray (for C-terminal domain of unknown function) or gray with pattern (for other domains with known function). When known, the corresponding domain number in databases is provided. TED related to HUH endonucleases are represented with different shades of cyan. HTH: Helix-Turn-Helix, wHTH: winged-HTH, SMC: Structural Maintenance of Chromosome (chromosome segregation protein). Note that the relaxases shown are representatives of given families, but some domains are not always present for other family members. (C) X-ray structures of TraI_Rel-F_ (Datta et al. 2003), MobA_Rel-R1162_ (Monzingo et al. 2007), and MobM_Rel-pMV158_ (Pluta et al. 2017). Catalytic residues are shown in pink, residues involved in bivalent ion coordination in orange, bivalent ion in gray. The DNA strands are labeled in black and gray.

Although considerable headway has been made toward definition of T4SS machine structures at high-resolutions in Gram-negative systems, structures of the relaxosome complexes required for initial processing of DNA substrates for transfer were only recently obtained. In a remarkable advance, structures have now been reported for the fully assembled relaxosome of the F plasmid from *E. coli* bound to various DNAs of its cognate *oriT* (Williams et al. 2025; Fig. 4A). The F relaxosome is made of TraI_Rel-F_, the relaxase belonging to the MOB_F_ family (Fig. 4B), and three auxiliary proteins, the plasmid-encoded TraY_Aux-F_ and TraM_Aux-F_ and the genomically encoded IHF. TraI is a bifunctional enzyme containing (i) a trans-esterase activity that acts on the *nic* site, and (ii) a helicase activity that acts to unwind the DNA in the 3′ direction. Interestingly, due to the requirement of a relaxase molecule also having to be transferred to the recipient cell to recircularize the ssDNA once transfer is completed, two relaxase molecules must bind to *oriT* on each side of the *nic* site (Ilangovan et al. 2017). A molecule binding 5′ of *nic* executes the nicking and covalent attaching at *nic*; as a result, this relaxase is transferred together with the ssDNA strand to the recipient cell. The second relaxase binds 3′ of *nic*, where it functions to unwind the DNA and remains in the donor cell. Williams et al. (2025) have now provided the first view of a fully assembled relaxosome, thereby elucidating the spatial arrangement of relaxosome components along *oriT* as well as their interactions with dsDNA, ssDNA, and each other (Fig. 2B, bottom panel). The new structures also define some of the *oriT* features required for function acquisition, notably the sequential involvement of the various domains during conjugation depending on DNA sequence and topology. Future perspective would be worth studying the relaxosome structure orchestrated by relaxases of other families.

### Relaxases of Gram-negative and -positive systems

The first conjugative relaxases studied at a molecular level were encoded by plasmids from *E. coli*. They included MbeA_Rel_, TraI_Rel-F_, TrwC_Rel_, and TraI_Rel-RP4_ from plasmids ColE1, F, R388, and RP4, respectively (Garcillan-Barcia et al. 2009). All of these relaxases belong to the large and widespread family of HUH endonucleases (Chandler et al. 2013). They are characterized by 3 conserved motifs, with motif I carrying the catalytic tyrosine, motif III harboring 3 histidine residues known to coordinate a bivalent cation required for the transesterification activity, and motif II whose role is still not elucidated (Ilyina and Koonin 1992). Based on phylogenetic analysis and on the conservation of these motifs, HUH relaxases have been classified into 5 different families: MOB_F_, MOB_P_, MOB_Q_, MOB_V_, and MOB_B_ (Garcillan-Barcia et al. 2009, Zechner et al. 2017). Figure 4B presents the domain architectures of these relaxase families, and Table 2 summarizes the main features of various prototypes of each relaxase family.

A number of differences among the different families can be noted. First, the MOB_F_ family harbors up to four tyrosine residues in motif I, with two being required for efficient transfer. By contrast, only one tyrosine residue is found in the active site of the other HUH relaxase families. A sub-family of the MOB_P_ family, exemplified by the MbeA_Rel-ColE1_, harbors a modified motif III with a HEN motif instead of the canonical 3H motif, thus corresponding to the MOB_HEN_ relaxases (Francia et al. 2004). Finally, the catalytic residue of motif I in MOB_V_ relaxases is not a tyrosine but a histidine residue (Pluta et al. 2017). Whereas the MOB_P_, MOB_V_, and MOB_Q_ families are found in MGEs from both Gram-negative and Gram-positive bacteria, MOB_F_ relaxases are rarely found in Gram-positive bacteria, and MOB_B_ relaxases are restricted to the *Bacteoridetes* phylum (Guglielmini et al. 2011). Experimental 3D structures are known for prototypes from several of these HUH relaxase families, including TraI_Rel-F_ and TraI_Rel-R1_ from the MOB_F_ family (Datta et al. 2003, Ilangovan et al. 2017), MobA_Rel-R1162_ from the MOB_Q_ family (Monzingo et al. 2007), and MobM_Rel-pMV158_ from the MOB_V_ family (Pluta et al. 2017) (Fig. 4C and Table 2). These structures confirmed that these families of relaxases are distantly related to each other, but nevertheless harbor the same fold as expected for HUH endonucleases (Chandler et al. 2013).

Besides these HUH relaxase families, a growing number of other relaxase families have been identified during the last 3 decades. Interestingly, each of them is distantly related to a different family of DNA metabolizing enzymes (Table 2). The MOB_H_ relaxases are encoded by large conjugative plasmids and ICEs, mainly from *Proteobacteria* (Garcillan-Barcia et al. 2009). They are related to HD-hydrolases. Only a single MOB_H_ relaxase has been characterized at the molecular level, the TraI_Rel-GGI_ encoded by the Gonococcal Genetic Island (GGI) from *Neisseria gonorrhoeae* (Heilers et al. 2019) (Fig. 4B). This GGI secretes ssDNA into the extracellular milieu, which can serve as a source of genetic variation upon acquisition by other *Neisseria* species harboring natural transformation systems (Callaghan et al. 2017). Two conserved motifs have been described: a 3H motif and a HD motif, both of which can coordinate divalent cations, probably Mn^2+^ and/or Co^2+^. Both motifs are required for the cleavage reaction at a site-specific sequence within *oriT*. SAXS experiments showed that the catalytic HD domain is globular, and that an additional C-terminal globular domain of unknown function is separated from the HD domain by an extended and disordered central domain (Heilers et al. 2019). Surprisingly, no stable covalent protein-DNA intermediate was detected. A trivial explanation is that experimental conditions for detection of such an intermediate have not yet been developed, but a more intriguing alternative is that MOB_H_ relaxases do not form such a covalent intermediate. If the latter, further studies are needed to determine how the DNA substrate is recruited to the T4CP, directed through the T4SS channel, and religated in the recipient cell.

Another non-canonical relaxase family is the MOB_C_ family, which is related to the PD-(D/E)XK superfamily of restriction endonucleases (Kosinski et al. 2005). MOB_C_ relaxases are encoded by conjugative plasmids and ICEs, as well as by mobilizable plasmids. They are found both in Gram-negative and in Gram-positive bacteria (Garcillan-Barcia et al. 2009, Guglielmini et al. 2011). A prototype of this family was studied experimentally, the TraX_Rel-pAD1_ from *Enterococcus faecalis* (Francia et al. 2013), and site-specific mutagenesis confirmed that the active site is related to that found in restriction endonucleases. Interestingly, however, TraX binds to the *oriT* at a *bind* site composed of 5 direct repeats that are located distantly from the *nic* site (about 70–120 bp downstream). Indeed, for most relaxases for which biochemical data are available, the *bind* site is generally close to the *nic* site (de la Cruz et al. 2010). As for MOB_H_ relaxases, no covalent intermediate with DNA was observed (Francia et al. 2013), raising the same questions about how the DNA is recruited to the T4SS, transferred, and religated in the recipient cell.

Although the MOB_T_ family of relaxases was identified decades ago, they were named only recently (Guglielmini et al. 2011). MOB_T_ relaxases are related to rolling-circle initiators of the *Rep_trans* family whose prototype is the RepC protein of the pT181 plasmid from *Staphylococcus aureus* (Novick 1989, Carr et al. 2016). They are mainly found in Gram-positive bacteria, more precisely in Bacillota within the Tn*916* superfamily of ICEs. They are also widespread in IMEs (integrative and mobilizable elements) from *Streptococcus* species (Coluzzi et al. 2017). Biochemical analysis of the active site of the MOB_T_ relaxase OrfJ_Rel_ from ICE*St3* (previously called RelSt3) confirmed this family is related to the *Rep_trans* proteins (Soler et al. 2019). As described for the MOB_C_ relaxases, the *bind* site of OrfJ_Rel-ICE_*_St3_* on *oriT* was shown to be located distantly from the *nic* site (about 70 bp) (Laroussi et al. 2022). However, it remains to be defined whether this is limited to OrfJ_Rel-ICE_*_St3_* or whether it is a general feature of MOB_T_ relaxases. Another interesting feature of MOB_T_ relaxases is that they are often encoded by ICEs or IMEs encoding a TcpA-like T4CP instead of the canonical VirD4-like T4CP (Ambroset et al. 2015, Coluzzi et al. 2017).

Finally, the most recently identified family of relaxases corresponds to the MOB_M_ family, which is related to tyrosine recombinases (Wisniewski et al. 2016). Its prototype is the TcpM_Rel_ relaxase of the pCW3 plasmid from *C. perfringens*. Whereas a tyrosine residue is required for DNA transfer *in vivo* and for relaxation of supercoiled plasmid *in vitro*, mutagenesis of other residues usually required for tyrosine recombinases had no effect (Wisniewski et al. 2016). TcpM_Rel_ protein is thus no longer a tyrosine recombinase but a *bona fide* relaxase, which was also demonstrated to bind *oriT* site-specifically. Given these different families of non-canonical relaxases, it appears that conjugative MGEs have succeeded in hijacking an impressive variety of proteins to play the role of relaxases to serve their propagation interest. With the discovery of numerous IMEs in Bacillota, evidence is accumulating that other protein families could also play the role of relaxases, such as the proteins that initiate rolling circle replication of the *Rep_2* family (Coluzzi et al. 2017). Clearly, the further exploration of IMEs and conjugative elements must be continued to obtain a complete catalogue of proteins that can be used as relaxases to catalyze MGEs transfer.

The transesterase domain of relaxases is generally not alone in carrying out the DNA processing steps orchestrated by relaxases. Indeed, relaxases are often multifunctional proteins (Garcillan-Barcia et al. 2009). The additional domains can assume various biochemical activities linked to DNA replication, as helicase or primase domains. MOB_F_ relaxases in particular are known to harbor a C-terminal helicase domain related to the superfamily I, which is required for transfer (Traxler and Minkley 1988). A specific cluster of MOB_Q_ relaxases also harbor a C-terminal helicase domain, whereas other MOB_Q_ relaxases have a C-terminal primase domain required for transfer (Henderson and Meyer 1999). Other domains can mediate DNA-binding, in particular at the *oriT bind* site. For example, OrfJ_Rel-ICE_*_St3_* harbors an N-terminal HTH domain that binds the *bind* site of its cognate *oriT* (Laroussi et al. 2022), whereas a winged HTH domain is found in the N-terminal part of TraX_Rel-pAD1_ (Francia et al. 2013).

### Relaxase auxiliary proteins

Auxiliary proteins are usually encoded by the MGEs but can sometimes be encoded by the cell, as when nucleoid-associated proteins are recruited. They often help the relaxase bind DNA, sometimes by bending DNA upon binding (Tsai et al. 1990, Rehman et al. 2019). They also often stimulate the nicking activity of the relaxase (Nelson et al. 1995, Ragonese et al. 2007). Among the best studied relaxosomal proteins are the TraY_Aux_ and TraM_Aux_ proteins of the F plasmid from *E. coli*. As noted above, host-encoded IHF was also shown to bind the F *oriT* (Tsai et al. 1990). The binding sites of these 3 auxiliary proteins are well mapped on the *oriT* DNA (see Fig. 4A), and their specific roles have been deciphered: they help TraI_Rel_ bind to *oriT* and also stimulate its nicking activity (Garcillan-Barcia et al. 2009). In numerous MOB_P_ systems, an auxiliary protein is needed for DNA transfer as it binds *oriT* whereas the relaxase is unable to do so. The auxiliary protein thus interacts with the relaxase and recruits it to the *oriT* for nicking. Examples of such auxiliary proteins exist in Gram-negative MOB_P_ systems, e.g. plasmid R64-encoded NikA_Aux_ recruits NikB_Rel_ to *oriT* (Yoshida et al. 2008), as well as in Gram-positive systems e.g. plasmid pCF10-encoded PcfF_Aux_ recruits PcfG_Rel_ to *oriT* (Rehman et al. 2019). This mechanism is also found in integrated elements, as with the ICE Tn*1549* and also with IMEs from Bacillota, where MobC_Aux_ recruits the MOB_P_ relaxase to *oriT* (Tsvetkova et al. 2010, Guedon et al. 2025). Most of the relaxosomal auxiliary proteins encoded by MGEs harbor a RHH (Ribbon-Helix-Helix) fold (Moncalian and de la Cruz 2004 et al. 2004, Yoshida et al. 2008, Varsaki et al. 2009, Godziszewska et al. 2014, Rehman et al. 2019). A notable exception corresponds to the HelP protein encoded by the ICE*Bs1* (which encodes a MOB_T_ relaxase). This HelP protein is a helicase processivity helper (Thomas et al. 2013), and it harbors an OB-fold (Oligonucleotide/oligosaccharide-binding fold) (Arcus 2002, Bianco 2022). Homologs encoded by ICE*St3* (namely OrfL_Aux1_ and OrfM_Aux2_) are OB-fold relaxosome auxiliary proteins that were also shown to modulate nicking-closing activities of OrfJ_Rel_ (Laroussi et al. 2025). This fold is also found in ssDNA binding (SSB) proteins involved in central DNA metabolism, exemplified by RPA in Eukarya and Archaea as well as single-stranded DNA binding proteins (SSBs) in Bacteria (Raghunathan et al. 2000, Yates et al. 2018, Madru et al. 2023).

## Functionally diverse and emergent systems

The foregoing discussion highlights the exciting advances made in recent years toward a detailed structural definition of T4SSs functioning in Gram-negative and Gram-positive bacteria. Notably, of the T4SSs structurally analyzed so far, all are assembled from a complete set of the core VirB and VirD4/TcpA subunits needed for translocation across the monoderm or diderm envelopes. Additionally, of the most extensively analyzed systems, all are thought to deliver discrete DNA fragments, e.g. MGEs, or effector proteins in one step across the bacterial cell envelope. There are, however, several T4SSs that rely on a subset of core VirB/D4 subunits to deliver novel substrate repertoires intercellularly or translocate substrates in two steps via a periplasmic intermediate. To gain a full understanding of the structural and mechanistic bases underlying such functional versatility, it is imperative that future studies expand the focus of detailed investigation to include such systems. Below, we briefly highlight a few systems, some known for decades but which remain understudied and others only recently identified, that we suggest merit special attention in the coming years.

### Systems lacking VirD4 homologs

**Figure 5.**
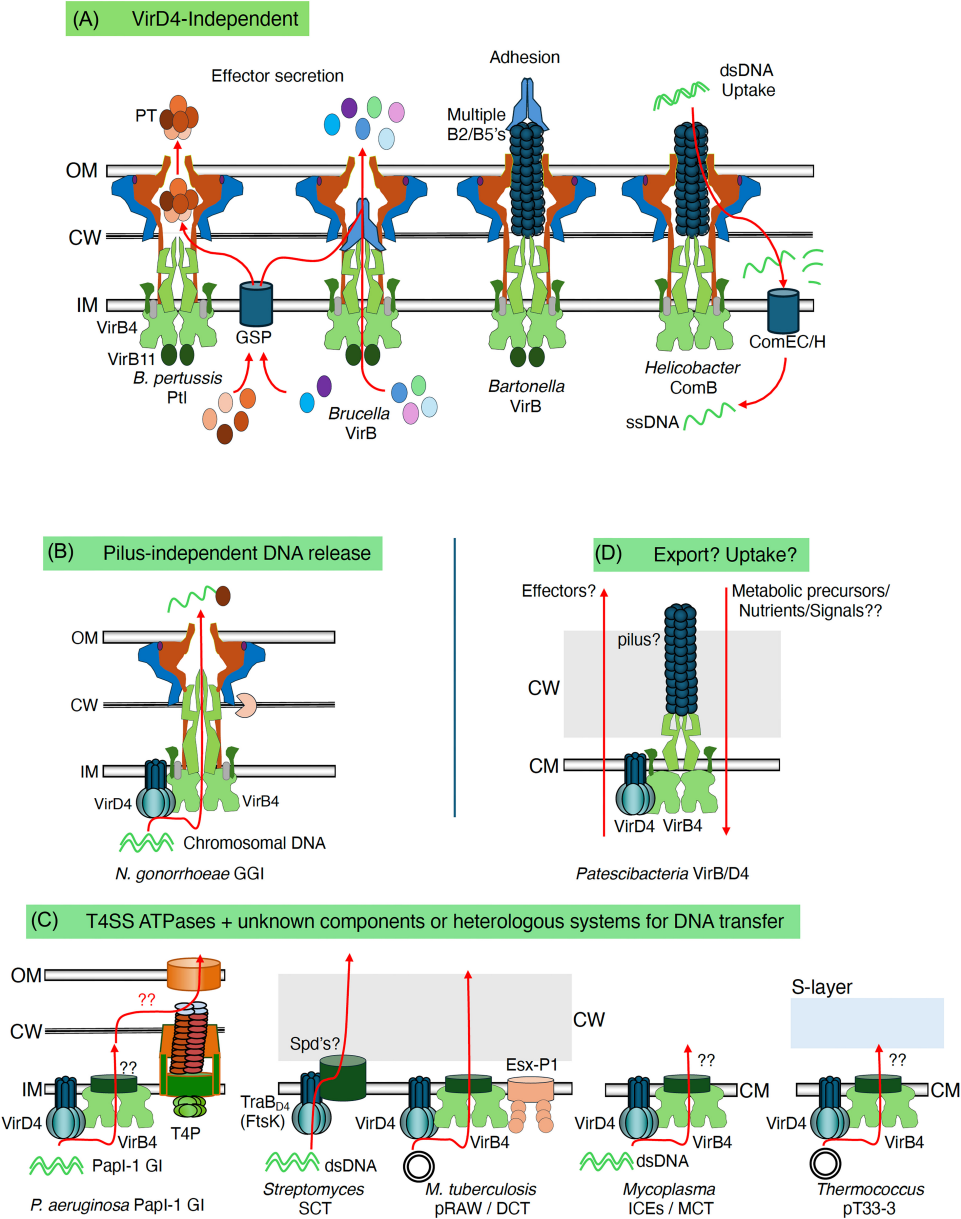
Functionally diverse T4SSs. (A) Systems lacking the VirD4-like T4CP can mediate effector release to the milieu or delivery to other cells via one-step or two-step translocation mechanisms, elaborate adhesive pili, or take up exogenous DNA. (B) The *N. gonorrrhoeae* GGI system releases DNA fragments into the milieu without a requirement for the TraA_B2_ pilin or F-like pilus. (C) The signature VirB4 ATPase and VirD4-like T4CP can function with currently unknown protein(s), a type IV pilus (T4P), or a type VII Esx system (T7SS) to conjugatively transfer plasmids (pRAW, pT33-3), ICEs (PapI-1 GI), or fragments of chromosomal DNA (termed SCT, DCT, MCT; see text). These systems deliver their substrates across diderm Gram-negative envelopes, thick cell walls or mycolic acid layers of monoderm Gram-positive envelopes, or S-layers of Archaeal species. (D) T4SSs of the recently described Patescibacteria are unique among the monoderm Gram-positive species in a) their requirement for growth of these epibiotic bacteria on their bacterial host species and b) their potential to elaborate conjugative pili. OM, outer membrane; IM, inner membrane; CW, cell wall; CM, cytoplasmic membrane; GSP, general secretory pathway. See text for other abbreviations and details.

Among the known Gram-negative systems, at least two convey protein substrates via a two-step translocation pathway. As mentioned earlier, when the T4SS name was first proposed, its members consisted only of the *A. tumefaciens* VirB and *B. pertussis* Ptl systems. The *A. tumefaciens* system emerged as an important paradigm for T4SSs that rely on the VirD4 T4CP to recruit and translocate DNA substrates in one step across the cell envelope. Strikingly, the *B. pertussis* system lacks the VirD4 receptor, and instead the PT subunits (S1-S5) are exported across the inner membrane via the general secretory pathway (GSP; Fig. 5A) (Weiss et al. 1993). In the periplasm, S1 through S5 assemble as the multisubunit PT, which is then recruited to the Ptl machine for export across the outer membrane (Farizo et al. 2000). The Ptl system also lacks a VirB5 tip pilus homolog, prompting a proposal that PT or one of its subunits serves as a structural analog for VirB5, enabling PT to dock onto the tip of a pilus-like structure (Weiss et al. 1993, Burns 2021). According to such a model, the pilus structure would dynamically extend and retract through the OMCC to reiteratively push PT complexes to the extracellular milieu. Compared with other protein substrates of T4SSs, which are thought to be translocated in unfolded or partially folded states (Trokter and Waksman 2018, Lettl et al. 2021), the size of assembled PT is huge, measuring 60 Å x 60 Å x 100 Å (Stein et al. 1994). Although the structure of the Ptl OMCC has not been solved, the VirB7, VirB9, and VirB10 homologs closely resemble the sizes and predicted domain architectures of their counterparts in the *A. tumefaciens* and pKM101 systems (Weiss et al. 1993, Farizo et al. 1996). Additionally, both the Ptl and *A. tumefaciens* VirB OMCCs can partially substitute for the equivalent pKM101-encoded substructure to support conjugative transfer of the pKM101 substrate (Gordon et al. 2017). How the large multiprotein PT is recruited to and delivered through the OMCC in the context of the Ptl machine assembled across the *B. pertussis* cell envelope remains an intriguing question.

Besides the Ptl system, T4SSs functioning in *Brucella* spp. also lack a T4CP (O’Callaghan et al. 1999) (Fig. 5A). To date, at least 15 secreted effectors have been identified, but the mechanisms of effector recognition and the temporal order of secretion during infection remain poorly understood (Myeni et al. 2013, Dehio and Tsolis 2017, Xiong et al. 2021). Although some effectors are recruited by the T4SS in the periplasm (Dohmer et al. 2014, Del Giudice et al. 2016), many do not possess signal peptides leaving the steps underlying their secretion unclear.

Another T4SS meriting further attention is that deployed by *H. pylori* to acquire DNA from the extracellular milieu. This system, designated ComB, is composed of homologs of VirB2-VirB4 and VirB6-10 (Hofreuter et al. 1998, Karnholz et al. 2006). Reminiscent of *B. pertussis* Ptl, this system lacks the VirD4 receptor dedicated to substrate secretion and the VirB5 pilus tip protein; it also lacks the VirB11 ATPase (Fig. 5A). The absence of VirB5 is of special interest insofar as VirB5 is implicated not only in adhesion of conjugative pili to target cells but also in initiation of pilus extrusion from the cell (Macé et al. 2022). Whether *H. pylori* elaborate pili to acquire dsDNA from the milieu remains to be determined. Regardless, by fluorescence microscopy, the ComB system was shown to function in exogenous dsDNA acquisition into the periplasm, and perhaps most remarkably, DNA uptake was blocked by pretreatment of cells with an ATP synthase inhibitor (Stingl et al. 2010). As ComB4_B4_ is the only ATPase associated with the ComB system, this latter finding suggests that a ComB structure—possibly a pseudopilus—serves as a DNA receptor at the cell surface and retracts by a mechanism dependent on the VirB4 ATPase. Rigorous tests of this retraction model are needed, because thus far the only T4SS shown to dynamically retract its pilus is that encoded by the F plasmid (Clarke et al. 2008, Goldlust et al. 2023). It is also noteworthy that the ComB system relies on a two-step translocation mechanism to import DNA into the bacterial cytoplasm. Once this system incorporates dsDNA into the periplasm, the DNA is converted to ssDNA and imported across the inner membrane via known components of other competence systems, ComH and ComEC (Stingl et al. 2010). The ComB system thus achieves dynamic two-step translocation by partnering with heterogeneous ComH/EC components to generate a functional chimeric machine.

Finally, *Bartonella* ssp. were shown to possess two distinct T4SSs required for infection, one assembled from VirB and VirD4 homologs and a second assembled only from VirB homologs. The VirB/D4 system delivers effector proteins into eukaryotic cells as a prerequisite for successful infection (Seubert et al. 2003). Remarkably, more recently, this system was also shown to deliver DNA substrates into eukaryotic cells, a process that is augmented when *Bartonella* cells are modified to additionally produce the R388 plasmid-encoded T4CP TrwB_D4_ (Fernandez-Gonzalez et al. 2011, Schroder et al. 2011). This latter finding suggests that R388 TrwB_D4_ functionally interacts with the *Bartonella* VirB/D4 system to enhance DNA transfer efficiencies. The second type of T4SS composed only of VirB homologs was termed Trw because the VirB components bear strong sequence relatedness to their counterparts in the plasmid R388-encoded Trw system (Fig. 5A). While it has not been excluded that the Trw T4SS, which lacks a VirD4-like T4CP, might secrete protein substrates via a two-step pathway analogous to that described for the *B. pertussis* Ptl and *Brucella* spp. VirB systems, it is noteworthy that the Trw T4SS possesses multiple sequence-variable copies of genes encoding the VirB2 and VirB5 homologs (Seubert et al. 2003). This has led to a proposal that the main function of the Trw system is to elaborate antigenically variable pili to enable *Bartonella* binding and infection of different host-specific erythrocytes (Seubert et al. 2003, Vayssier-Taussat et al. 2010). For this latter model, the Trw T4SS would have appropriated the pilus-producing function of conjugation systems while dispensing with the associated translocation function. Whether the Ptl, ComB, and Trw systems indeed elaborate pili or pseudopili and whether these pili dynamically extend and retract are important questions for future work.

### Noncanonical systems functioning to translocate chromosomal DNA fragments

T4SSs or chimeric systems composed of T4SS-like subunits and other subunits of different ancestries have also evolved to translocate large chromosomal fragments rather than MGEs of discrete sizes. One system, encoded by the GGI harbored by *N. gonorrhea* (Dillard and Seifert 2001, Hamilton et al. 2005), resembles the *E. coli* Hfr strain discovered by Lederberg and Tatum in 1946. Like Hfr, the *N. gonorrhoea* GGI carries an F-like transfer system, but remarkably functions to deliver chromosomal DNA to the extracellular milieu rather than directly to another bacterial cell (Fig. 5B). The early substrate processing reactions are similar insofar as the GGI encodes a relaxase that nicks at a specific *oriT* site within the GGI to initiate transfer of a single strand of chromosomal DNA. However, the GGI F-like machine does not require donor-recipient cell contact for activation, resulting in ssDNA secretion instead of intercellular transfer. *Neisseria gonorrhoea* is naturally competent, and therefore the release of chromosomal DNA supplies neighboring *N. gonorrhoea* cells with exogenous DNA as a source of genetic variation (Ramsey et al. 2011). Intriguingly, the GGI system does not require the TraA_B2_ pilin subunit to achieve DNA export (Pachulec et al. 2014). In nearly all Gram-negative T4SSs characterized to date, a pilin or pilin-like subunit is an essential component of the transfer apparatus, even if the systems naturally or by mutation fail to elaborate pili. What types of structural adaptations has the GGI F-like system evolved to achieve contact-independent DNA translocation in the absence of the pilin or extracellular pilus?

In *Pseudomonas aeruginosa*, the PapI-1 genetic island (GI) encodes a novel DNA transfer system that formally resembles the conjugation systems of IncI plasmids such as R64, but with intriguing adaptations (Fig. 5C) (He et al. 2004, Foley et al. 2021). The IncI plasmids carry two gene clusters contributing to plasmid transfer. One encodes a set of Dot/Icm-like homologs and is considered the progenitor conjugation system from which the *L. pneumophila* Dot/Icm system evolved. The second encodes a type IV pilus (T4P), which is elaborated by a type II-like secretion machinery and is completely unrelated to the conjugative pilus elaborated by T4SSs. For IncI plasmids, the Dot/Icm-like T4SS is required for transfer under all mating conditions, whereas the T4P augments transfer in dilute, liquid environments presumably by promoting contacts between donor and physically distant recipient cells (Foley et al. 2021). The PapI-1 GI carries genes for the type IV pilus, but in this case the T4P is essential for DNA transfer under all mating conditions (Fig. 5C) (Carter et al. 2010). Furthermore, the PapI-1 GI additionally codes for only two clear T4SS homologs, which correspond to the signature VirB4 ATPase and the VirD4 T4CP (Carter et al. 2010). One model posits that the PapI-1 system evolved as a chimeric system in which VirB4 and VirD4 orchestrate delivery of the Pap1-I substrate across the inner membrane and then coordinate with the T4P to deliver the substrate across the periplasm/OM of the donor and into the recipient cell. Such a model not only predicts a completely novel translocation pathway across the donor cell envelope, but also a novel function for a T4P in mediating the delivery of a DNA substrate to another cell. It should be noted that the PapI-1 GI also codes for hypothetical proteins with predicted features reminiscent of T4SS subunits (membrane insertion, lytic transglycosylase, lipoprotein) (Carter et al. 2010). Whether these hypothetical proteins comprise components of a functional equivalent of the canonical T4SS channels remains to be tested. Even at this juncture, the essentiality of the T4P for PapI-1 transfer establishes this system as a novel type of conjugative T4SS.

In Gram-positive bacteria, several systems stand out as DNA translocators with distinctive properties. As noted earlier, T4CPs are ancestrally related to the FtsK and SpoIIIE ATPases, which function as dsDNA translocases (Becker and Pogliano 2007, Bigot et al. 2007, Cabezon et al. 2012). An intriguing feature of FtsK and closely related hexameric ATPases is that they use a revolving mechanism to unidirectionally transport genomic dsDNA across membranes (Weitao et al. 2023). As dsDNA extends through the central channel of the hexamer, one functional subunit of the DNA translocase contacts the DNA at a time. ATP hydrolysis induces conformational changes in the translocase domain, which delivers the DNA backbone to the next functional unit inside the hexamer by a sequential hand-off mechanism. Such revolving action has not been demonstrated for T4CPs associated with the well-characterized T4SSs but is strongly implicated for the plasmid conjugation machine TraB functioning in *Streptomyces* (Weitao et al. 2023). The TraB hexamer is phylogenetically and structurally very similar to FtsK and SpoIIIE, and it has been shown to deliver dsDNA forms of conjugative plasmids, actinomycetes integrative and conjugative elements, or chromosomal DNA across *Streptomyces* mycelial membranes (Vogelmann et al. 2011, Thoma and Muth 2016). TraB can thus be considered to be a T4CP that functions independently of other known T4SS subunits (Fig. 5C). However, all *Streptomyces* conjugative plasmids carry *spd* genes, often organized as transcriptional units along with *traB*. Encoded Spd proteins insert into the membrane and are required for intramycelial dsDNA transfer, suggestive of a coordination of function with TraB_D4_ (Thoma et al. 2015). Whether a TraB/Spd complex assembles analogously to that of the VirD4/T4SS complexes of the canonical systems awaits further study. TraB mediates DNA transfer through binding of 8-base pair (bp) repeats termed the *cis*-acting-locus (*clt*) carried by conjugative plasmids as well as *clt*-like sequences distributed throughout *Streptomyces* chromosomes (Vogelmann et al. 2011). Remarkably, a recent study demonstrated that a single conjugation event can result in replacement of between 1.5 and 37.5% of the recipient genome, distributed in fragments across the chromosome (Choufa et al. 2024). Although the mechanism underlying TraB-dependent *Streptomyces* chromosomal transfer (SCT) remains to be determined, such large-scale genomic shuffling is proposed to aid in adaptation of *Streptomyces* populations, resulting in broadened metabolic capabilities, e.g. new antibiotics or increased antibiotic yields, niche differentiation, and on a longer timescale species diversification (Choufa et al. 2024).

Distinctive mechanisms of conjugative DNA transfer also exist among the Mycobacteria. One system, identified in the fast-growing (2–4 h doubling time) mycobacterial species *Mycobacterium smegmatis*, accounted for early findings that *M. smegmatis* donors transfer chromosomal auxotrophic marker mutations to recipients in prolonged solid-surface matings (Wang et al. 2003). Although formally reminiscent of *E. coli* Hfr transfer, several remarkable differences exist (Wang et al. 2005). In Hfr transfer, the donor chromosomal genes are transferred with efficiencies correlating with their distance from the integrated *oriT* sequence. By contrast, in *M. smegmatis* donor chromosomal genes are transferred with equal efficiencies regardless of their location. More recent detailed analyses revealed the surprising finding that, while selectable marker genes of interest were transferred during mating as expected, so also were many other unlinked and unselected donor DNA segments (Gray et al. 2013, Derbyshire and Gray 2014). On average, transconjugant genomes contained 575 kb of donor DNA in 13 segments of sizes ranging from 59 to 226 kb. These transferred segments were distributed around the chromosome giving rise to a mosaic transconjugant genome composed of DNA tracts from both parents; the underlying process was thus termed distributive conjugal transfer (DCT) (Gray et al. 2013, Derbyshire and Gray 2014). Although the outcome of DCT formally resembles that described for distributive chromosomal transfer in *Streptomyces*, the machinery mediating DCT in *M. smegmatis* is quite distinct from TraB-mediated transfer through recognition of *cis*-acting *clt* sequences. Remarkably, this machinery is encoded by a class of genes associated with the ESX secretion systems, which are also known as type VII secretion systems (T7SSs) (Bitter et al. 2009). The *esx* loci are composed of ∼10 core genes (ecc’s for esx conserved component) that code for a membrane transporter, chaperones, and ATPases. In *Mycobacterium tuberculosis*, the ESX systems export proteins required for virulence (Groschel et al. 2016), whereas in *M. smegmatis* ESX-1 and ESX-4 are required for DCT (Gray et al. 2016). ESX-4 is of particular interest. It is a paired-down ESX system, composed only of 7 genes and missing several required for function of other ESX’s (Groschel et al. 2016). But most intriguingly, *esx4* mutations in donor cells have no impact on DCT, whereas equivalent mutations in recipient cells completely abolish DCT (Gray et al. 2016). The donor and recipient *esx* systems in donor and recipient cells are essentially identical, suggesting that this system functions in a specific cellular context, possibly by directing DNA uptake as opposed to DNA export. Adding to the complexity of this mating system, ESX-1 mutations in donor cells confer a hyper-conjugative phenotype, whereas the equivalent mutations in recipient cells block DCT (Gray et al. 2013, Gray et al. 2016). ESX-1 appears to function by regulating *esx4* gene expression in the recipient; when *esx1* mutations exist in the recipient cell, they impair *esx4* expression but when *esx1* mutations exist in the donor cell, they confer hyperexpression of *esx4* in the recipient, resulting in the hyper-conjugative phenotype. These latter findings have prompted a proposal that *esx1* mediates intercellular communication from recipient to donor cells via propagation of a molecular signal (Gray et al. 2016).

The ESX/T7SSs systems are phylogenetically and mechanistically distinct from the T4SSs; however, their capacity to promote DNA transfer functionally aligns the ESX-1/ESX-4 systems of *M. smegmatis* with the canonical conjugation machines. In fact, this alignment is more than just a case of convergent evolution in which two distinct ancestral protein translocators (the progenitors of T4SS/conjugation and T7SS systems) evolved to recognize and translocate DNA substrates. Researchers established that conjugative plasmids in the slow-growing mycobacterial species *M. tuberculosis* carry not only a T4SS locus but also a previously unrecognized T7SS locus (Ummels et al. 2014). Mutational analyses further confirmed that both loci are required for conjugation, establishing that both secretion systems, possibly functioning as a chimeric system, mediate DNA transfer (Fig. 5C). Notably reminiscent of the PapI-1 system of *P. aeruginosa*, the T4SS locus carries genes for the core VirB4 ATPase and VirD4 T4CP subunits as well as several hypothetical proteins (Ummels et al. 2014). Whether the core VirB4/VirD4 complex assembles with these hypothetical proteins to build a functional translocation channel or instead engages with the T7SS system to build a chimeric DNA translocation system is unknown. That a conserved VirB4/T4CP complex has adapted on the one hand to functionally interact with a T4P in a Gram-negative species and on the other a T7SS in a Gram-positive species to promote DNA transfer highlights the extreme versatility of T4SSs and their components for the evolution of novel functions.

The discovery of ICEs in genome-reduced mycoplasmas has raised fundamental questions about the architecture and functioning of conjugative systems in these wall-less bacteria (Marenda et al. 2006). ICEs identified in *Mycoplasma agalactiae* and *M. bovis* have been shown to mediate the transfer not only of their own genetic material but also of large chromosomal regions from the donor to recipient cells, a process referred to as mycoplasma chromosomal transfer (MCT) (Fig. 5C) (Dordet Frisoni et al. 2013, Tardy et al. 2015). This mechanism plays a pivotal role in genome plasticity and accelerates the evolution and adaptation of these minimal bacteria. Attempts to identify homologs of canonical VirB-type conjugation machinery revealed the presence of five ICE-encoded genes, CDS5 and CDS17, which encode for the VirD4 and VirB4 homologs, respectively, CDS16, which encodes for an integral membrane protein with eight transmembrane helices and is likely the VirB6-like component of the system, and CDS13 and CDS15, which encode proteins with features similar to VirB3 (Baranowski et al. 2018, Derriche et al. 2025). The absence of VirB1-like and VirB8-like components suggests that mycoplasma ICEs rely on a minimal and highly adapted conjugation apparatus functioning in the unique membrane physiology of these bacteria.

### Gram-positive systems with other novel functions

Given the plethora of functions ascribed thus far to T4SSs in Gram-negative bacteria (conjugation, effector translocation, DNA export, DNA import, toxin protein release, interbacterial toxin delivery), it is surprising that nearly all known T4SS functions in Gram-positive bacteria are limited to unidirectional DNA translocation. The question of whether T4SSs in the Gram-positives have evolved as dedicated effector translocators to aid in various infection processes is a long-standing one and requires considerably more attention. In fact, over a decade ago, researchers presented strong evidence for effector secretion by a T4SS encoded by the 89 K pathogenicity island (PAI) of *Streptococcus suis* 2 (Zhao et al. 2011). The 89 K PAI, which is responsible for the highly invasive virulence phenotype of *S. suis* 2, carries a T4SS locus with the signature genes for six core VirB and VirD4 subunits (VirB1, VirB3, VirB4, VirB6, VirB8, VirD4) harbored by most MGEs in Gram-positive species (Zhao et al. 2011). Confirming the functional importance of the T4SS locus, a Δ*virD4* T4SS-inactivating mutation strongly attenuated *S. suis* virulence (Zhao et al. 2011). More recently, the T4SS was shown to secrete a subtilisin-like serine protease, SspA-1, to the extracellular milieu (Yin et al. 2016). A Δ*sspA-1* mutation phenocopied the Δ*virD4* mutation, resulting in highly attenuated virulence in mouse models. The 89 K PAI-encoded system thus appears to constitute the first effector translocator of the T4SS superfamily operating in a Gram-positive species. The findings raise two important questions, how does this T4SS achieve protein export across the Gram-positive cell envelope, and do other T4SSs export effectors to the milieu or into other cells?

Finally, perhaps the most exciting newcomers to the T4SS superfamily are systems found in a large group of monoderm bacteria designated as Patescibacteria. This phylum of bacteria, also known as the candidate phyla radiation (CPR), constitutes a highly diverse group of ultrasmall (100–300 nm width) bacteria found in various environments as well as humans (Coleman et al. 2021). The phylum was identified originally through metagomic DNA sequencing and constitutes a significant fraction of microbial dark matter, a name given to the collection of Bacterial and Archaeal species that have not been cultivated (Marcy et al. 2007). Recently, researchers showed that Saccharibacteria, a group of human oral cavity-associated Patescibacteria (Bor et al. 2019), could be cultivated in the lab by growth on Actinobacteria host cells (He et al. 2015). As shown for other members of Patescibacteria, the Saccharibacteria genomes are small (typically < 1 Mb) and devoid of genes for the respiratory chain and pathways for de novo synthesis of amino acids, nucleotides, and fatty acids. However, they do carry genes for various secretion systems, including T4SSs, T2SSs, and T4P (He et al. 2015, Wang et al. 2023, Quinonero-Coronel et al. 2025). By use of T4P inhibitors, evidence was presented that T4Ps mediate twitching motility and adhesion to bacterial host cells, and contribute to epibiotic growth (Xie et al. 2022). The T4Ps also were shown to confer natural competence, allowing for the first time the genetic manipulation of species within the CPR (Wang et al. 2023). Capitalizing on this advance, large-scale Tn-seq analyses established the essentiality of both the T4P and the T4SS gene clusters for survival of Saccharibacterial species *Southlakia epibionticum* ML1 (designated *Se*) during co-culture with its host *Actinomyces israelii* (Wang et al. 2023). This species also carries a competence locus that includes *comEC*, and as expected a Δ*comEC* mutation abolished competence. However, this mutation did not affect *Se* viability, showing that natural transformation does not serve as an essential mechanism for acquisition of nucleotides for DNA synthesis. This finding also establishes that essentiality of the T4P and T4SS machines cannot be due to functional interactions of one or both machines with ComEC (Wang et al. 2023).

The Se T4SS is the first described T4SS shown to be essential for survival of the host bacterium. As *Patescibacteria* are monoderms, it is not surprising that the T4SS locus codes for proteins characteristically associated with T4SSs of other Gram-positive bacteria, *e.g*. VirB1-, VirB3-, VirB4-, VirB6-, VirB8-, and VirD4-like components (Wang et al. 2023, Quinonero-Coronel et al. 2025). Remarkably, this locus also carries a pilin gene cluster encoding 6 structurally conserved, but sequence-divergent VirB2-like pilins (Fig. 5D) (Wang et al. 2023, Quinonero-Coronel et al. 2025). This pilin array is essential for *Se* survival, raising the intriguing possibility that a T4SS functioning in a Gram-positive species elaborates a pilus-like structure. If so, it is enticing to speculate that the T4SS and extracellular pilus act as a conduit for exchange of signals or uptake of various molecules from the *A. israelii* host cell to support *Se’s* epibiotic lifestyle.

### Achaeal systems

T4SSs also act to transfer DNA in Archaea. Conjugation was first discovered in *Crenarchaea* during the 90’s, especially in the *Sulfolobus* genus, which are thermo-acidophilic archeaons. The first plasmid demonstrated to be conjugative was pNOB8 from a Japanese isolate (Schleper et al. 1995). Subsequently, several other conjugative and mobilizable plasmids were isolated from Iceland (Stedman et al. 2000, Erauso et al. 2006). They are 25–45 kb in size, and most of them have high copy numbers and trigger growth retardation. Interestingly, only 2 conserved genes encode proteins displaying significant sequence similarities with proteins involved in bacterial T4SS: VirB4 and VirD4 homologs. Another intriguing conserved protein is encoded in the same cluster of genes; it is a membrane protein harboring significant similarities with permeases as well as a domain conserved among Smc (structural maintenance of chrosomomes) proteins. These three proteins thus might form a membrane complex capable of pumping DNA across the archaeal envelope and into the recipient cell (Erauso et al. 2006). More recently, the first case of conjugation in the phylum *Euryarchaea* was demonstrated with the pT33-3 plasmid from a strain of the hyperthemophilic genus *Thermococcus* (Catchpole et al. 2023). This 103 kb plasmid also encodes only VirB4- and VirD4-like proteins (Fig. 5C). Mutation of the corresponding genes abolished interspecies plasmid conjugation, demonstrating for the first time their involvement in archaeal conjugation. Surprisingly, an interphylum transfer of pT33-3 was demonstrated from the original euryarchaeon to a crenarchaeon, indicating this plasmid could be a broad-host-range plasmid. Up to now, no relaxase has been identified on these plasmidic genomes based on sequence similarities. These fascinating findings underscore the need for further detailed investigations aimed at deciphering molecular mechanisms of conjugation in Archaea.

## Summary and future directions

The remarkable versatility and diversity of T4SSs underscore the need of a unifying nomenclature to guide future discussion and enable meaningful comparisons. Comparative analyses of Gram-negative and Gram-positive systems have revealed both shared architectural aspects and distinctive features, reflecting the adaptability of these molecular machines. This adaptability is further emphasized by the hybrid assemblies, in some cases integrated with the type IV pili, ESX systems, or other cell surface complexes. Despite the recent advances in structural definition of these systems, key questions remain unresolved, including the determinants of cross-system compatibility and the evolutionary pressure shaping their diversification. Addressing these knowledge gaps will not only refine our understanding of these dynamic machines but may also open novel strategies for engineering chimeric systems with tailored functions.
